# An *in vitro* assay and artificial intelligence approach to determine rate constants of nanomaterial-cell interactions

**DOI:** 10.1038/s41598-019-50208-x

**Published:** 2019-09-26

**Authors:** Edward Price, Andre J. Gesquiere

**Affiliations:** 1 NanoScience Technology Center, University of Central Florida, Orlando, FL 32826 USA; 2 Department of Chemistry, University of Central Florida, Orlando, FL 32816 USA; 3 Department of Materials Science and Engineering, University of Central Florida, Orlando, FL 32816 USA; 4 The College of Optics and Photonics (CREOL), University of Central Florida, Orlando, FL 32816 USA

**Keywords:** High-throughput screening, Virtual drug screening, Cell-particle interactions

## Abstract

*In vitro* assays and simulation technologies are powerful methodologies that can inform scientists of nanomaterial (NM) distribution and fate in humans or pre-clinical species. For small molecules, less animal data is often needed because there are a multitude of *in vitro* screening tools and simulation-based approaches to quantify uptake and deliver data that makes extrapolation to *in vivo* studies feasible. Small molecule simulations work because these materials often diffuse quickly and partition after reaching equilibrium shortly after dosing, but this cannot be applied to NMs. NMs interact with cells through energy dependent pathways, often taking hours or days to become fully internalized within the cellular environment. *In vitro* screening tools must capture these phenomena so that cell simulations built on mechanism-based models can deliver relationships between exposure dose and mechanistic biology, that is biology representative of fundamental processes involved in NM transport by cells (e.g. membrane adsorption and subsequent internalization). Here, we developed, validated, and applied the FORECAST method, a combination of a calibrated fluorescence assay (CF) with an artificial intelligence-based cell simulation to quantify rates descriptive of the time-dependent mechanistic biological interactions between NMs and individual cells. This work is expected to provide a means of extrapolation to pre-clinical or human biodistribution with cellular level resolution for NMs starting only from *in vitro* data.

## Introduction

**Figure 1:**
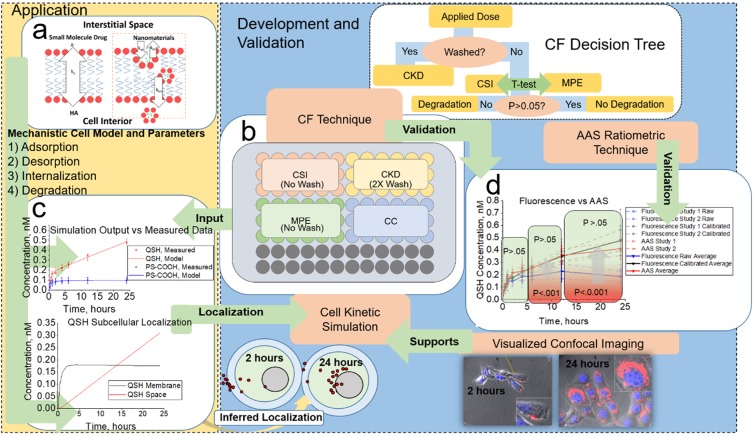
Schematic overview of FORECAST. The CF assay is coupled to an *in vitro* kinetics simulation. (**a**) Traditional partition coefficients (k_p_) commonly used for small molecule partitioning between blood supply and cell interior. For NMs, rate constants for adsorption (k_ad_), desorption (k_de_), internalization (k_int_), and degradation (k_deg_) more accurately represent uptake processes incident on a NM when exposed to cell environment. (**b**) Layout of CF assay, including CSI compartment (internal standard and descriptive of cellular degradation), CKD compartment (descriptive of kinetics of NM-cell interactions), MPE compartment, (descriptive of media degradation), and CC compartment (descriptive of control with cells without NM exposure). The CF decision tree illustrates how these are connected in the assay. CF outputs were then used to build a simulation (**c**) with parameters descriptive of adsorption, desorption, internalization, and degradation pathways. Data from CF was also (**d**) validated to AAS outputs.

Nanomaterial (NM) applications span across fields such as medicine^1,2^, electronics^3^, materials development^4,5^, and agriculture^6^. Increased NM development increases the chance of exposure to these materials and brings the need for robust high-throughput methodologies to assess and quantify NM biodistribution accurately^7–9^. Current methods to determine NM *in vivo* biodistribution often require animal sacrifice and tissue resection for further processing^10^. This process is time-consuming and requires substantial resources, as often only one time point is available per animal^11^. Simulation-based approaches that incorporate animal physiology^12^, such as physiologically based pharmacokinetic models (PBPK), are a possible solution to this problem as they have proved successful for small molecules^13^. Traditionally, these simulations assume immediate diffusion of drug from blood to whole tissue based on partitioning coefficients, k_p_^14,15^. NMs, however, do not undergo immediate diffusion unless they are ultra-small^16–18^. Instead, they interact with individual tissue cells through active transport processes such as e.g. vesicular transport through endocytic or phagocytic pathways^19^ (Fig. 1a). Thus, general tissue-blood partitioning (k_p_) is not sufficient to describe NM-biological interactions. Current NM PBPK approaches also rely heavily on estimation of k_p_ from experimental animal data^20–22^. In recent NM PBPK simulations, k_p_ has been replaced with a combination of endothelial penetration (optimized from animal data), total macrophage uptake (obtained *in vitro*), and macrophage/endothelium release rates (optimized from animal data)^23^. These modifications resulted in inclusion of time-dependent NM transport from the blood supply into tissue cells. However, macrophage rates were determined from both *in vitro* to *in vivo* extrapolation of intrinsic clearance (mL/min/cell) and optimization of total macrophage release rates to animal data, and critical mechanisms involved in NM transport (adsorption, desorption, internalization)^24^ remain unaccounted for.

To achieve *in vivo* predictive capabilities for NMs, animal simulations must utilize mechanistically informed kinetics, that is, kinetics representative of biological processes involved in NM uptake (for example transport to the cell membrane and cell interior, which can take up to 24 hours in some cases^25,26^). This information can be obtained *in vitro*, but has been difficult to acquire with techniques for high-throughput analysis^27,28^, and in many cases internalized and membrane-bound particles^29^ are difficult to distinguish through routine analysis (although efforts have been made to determine this experimentally^30^). Moreover, cell-induced degradation^31^ of NMs is often overlooked^28,32–34^, which can lead to misinterpretation of the true exposure dose^27^ when fluorescence is used as an indicator of number of particles^35^. *In vitro* methods could help to address these knowledge gaps, but so far there exists a limited connection between *in vitro* quantitative outputs and *in vivo* biodistribution. The reason is that data obtained in cell and animal studies are primarily observational quantities acquired from tissue homogenization. Different studies also tend to be based on different methods that are difficult to quantitatively correlate, for example flow cytometry and atomic absorption spectroscopy (AAS). *In vitro* methods that account for these limitations are key if *in vitro* data is to be useful for extrapolation to animal biodistribution and hazard assessment. To help bridge the gap between *in vitro* cell uptake results and *in vivo* biodistribution^7,8,13,27^, a combination of high-throughput *in vitro* quantitative methods and predictive computational models could be used.

Here, we report FORECAST (Fluorescence Cell Assay and Simulation Technique), a method in which we utilize a high-throughput quantitative fluorescence *in vitro* assay (Fig. 1b) coupled with a cell simulation (Fig. 1c) that uses an artificial intelligence-based algorithm^36^. FORECAST delivers rates descriptive of NM adsorption to and from the cell membrane, internalization, and degradation.

The *in vitro* fluorescence assay takes into account effects of cell-induced and media-induced degradation through calibrated fluorescence analysis (calibrated fluorescence, or CF) and was validated by Atomic Absorption Spectroscopy (AAS, Fig. 1d). Here we demonstrate the capabilities of the CF assay to account for NM degradation through the use of degradable quantum dots (QSH)^32,37^, and compare the results with stable dye loaded polystyrene NMs (PS). To outline the overall FORECAST method, data from the *in vitro* CF assay feeds directly to the cell simulation to extract rates descriptive of cellular adsorption, desorption, internalization, and degradation. Thus, the FORECAST approach gives vital information on (1) NM stability, (2) quantitative kinetics of cellular uptake, and (3) localization on cells (membrane or internal cell space) that can be transferred to *in vivo* models. The *in vitro* rates for tissue cells obtained from FORECAST will, in the future, extrapolate to *in vivo* simulations for the development of more predictive animal models (Supplementary Fig. 1). This could provide an avenue towards reduction of animal testing.

## Results

### *In vitro* assay design

The fluorescence signal obtained in the assay quantifies NM uptake by cells while giving insight in cell or media induced degradation. Negatively charged 14 nm QSH (CdSe/ZnS carboxy-functionalized quantum dots) and 36 nm PS (carboxy-functionalized dye loaded polystyrene nanoparticles) are utilized here (characterization in Supplementary Table 1) due to their known degradation and stability *in vitro*, respectively^38,39^. Hepa1-6 was chosen as the model cell line to demonstrate the method because previous studies show significant NM in the liver.^40^

The CF assay (schematic in Fig. 1b) is built starting from the application of cells on a 96-well plate in 3 “compartments”: the Cell System Interactions (CSI) compartment (*cells* *+* *NM (unwashed)*, *accounts for cell-induced NM degradation*), the Cell Kinetic Data (CKD) compartment (*cells* *+* *NM (washed)*, *measurement of NM uptake*), and the Cell Control (CC) compartment (*cells in media* *+* *no NM (unwashed)*, *control with untreated cells to subtract background signal*). The Media and Protein Effect (MPE) compartment (*no cells* *+* *NM in media (unwashed)*) accounts for media and protein induced degradative effects on the NM in the absence of exposure to cells. Note that the CSI and MPE compartments are never washed and therefore maintain the initial applied dose of NM (10 nM in this demonstration of the method). The CKD compartment is washed at each time, *t*, to remove NMs that are not cell membrane bound or internalized by cells. Control experiments on blank wells showed minimal NM adhesion to the sides and surface of wells, indicating all fluorescence should strictly come from NM interacting with cells. For the CF assay development and validation to AAS (for QSH), we used 18 wells per compartment, which resulted in one 96-well plate per time point. In applications of the method, such a large statistical group is not required. Therefore, wells will be freed up to allow for multiple time points and cell types on the same plate.

We allowed cells to reach 90% confluence and establish membrane integrity^41^ after 48 hours. Comprehensive analysis on toxicity (Supplementary Fig. 2) and stability (Supplementary Fig. 3) reveal minimal toxicity and sedimentation for a 10 nM dose of the NMs chosen for this study, supporting assumptions of minimal cell detachment and constant exposure dose (minimal sedimentation effects)^42^. All data are within the linear dynamic range, as well as within the limits of detection and quantitation (Supplementary Fig. 4, Supplementary Table 2).

**Figure 2:**
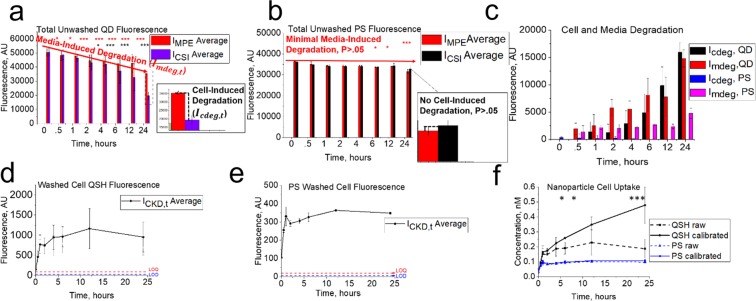
Fluorescence data from cell kinetic studies for 0, .5, 1, 2, 4, 6, 12, and 24 hours. Total fluorescence for wells with (CSI) and without (MPE) cells exposed to 10 nM for (**a**) QSH and (**b**) PS. Fluorescence intensities I_CSI,t_ and I_MPE,t_ for (**a**) differ significantly (P < 0.05) for QSH at approximately 4 hours, indicating cell-induced cellular induced degradative effects. Fluorescence intensities I_MPE,t_ and I_MPE,0_ differ significantly from 30 minutes, indicating media degradation at this time. For PS (**b**), no significant cell induced degradation is present (P > 0.05). Media induced degradation begins at 6 hours (P < 0.05). Differences between I_CSI,t_ and I_MPE,t_ and I_MPE,t_ with I_MPE,0_ were plotted in (**b**), showing a steady increase for QSH and constant intensity for PS. Media (I_mdeg_) and cell (I_cdeg_) induced degradative values were calculated from (**a**, **b**) and plotted against time in (**c**). Fluorescence intensities were taken after washing 2X with complete growth medium and further trypsinization for (**d**) QSH and (**e**) PS. Cell fluorescence values from CKD (**d**,**e**) were ally used to determine concentration (nM) of QSH and PS NM uptake/adsorption in (**f**). The calibrated concentration of QSH (black, solid) shows significantly higher values (P < 0.05) than raw values (black, dashed). However, PS does not degrade, so calibrated (blue, solid) and raw (blue, dashed) concentrations are not significantly different. Statistical t-test p-values are described as follows: ***P < 0.001, **P < .01, and *P < 0.05.

At time zero, the CSI, CKD, and MPE compartments were dosed with 10 nM QSH or PS (10% FBS DMEM suspension), with one NM type per plate. Comparing (by t-test) the fluorescence signal for wells in the CSI compartment at time *t* with the fluorescence signal from wells in the MPE compartment at time *t* gives insight into cell-induced degradation (see Fig. 1 CF decision tree). If they are statistically different we conclude cell induced NM degradation is present and the quantity of fluorescence signal loss due to this effect is determined from the difference of CSI and MPE at time *t* (Fig. 2a–c). Here, this effect is suggested to represent an irreversible (Supplementary Fig. 5) and total loss of fluorescence of a fraction of the internalized NMs (due to lysosomal degradation), rather than a partial loss of fluorescence of a larger portion of the NMs as a result of their interactions with the cell membrane or intracellular environment.

Similarly, comparing the fluorescence signal (by t-test) from wells in the MPE compartment at time *t* with respect to MPE at time zero gives a description of media-induced degradation. These critical steps guide NM uptake calculations, especially if degradation is present.

Fluorescence data on NM-cell total uptake (adsorbed + internalized) were obtained from the CKD compartment after washing 2X, trypsinizing, and mixing (Fig. 2d,e), with minimal trypsin interference on signal found (Supplementary Fig. 6).The CKD fluorescence signal at time *t* (*I*_*CKD*,*t*_) is representative of adsorbed and internalized NM. Comparison of signal obtained from CKD to CSI at time *t* (*I*_*CSI*,*t*_) for a given concentration of NM applied (nM, [Dose]) will yield the calibrated concentration of NM taken up by cells ([*Uptake*]_c,t_) as follows,

1 $${[Uptake]}_{c,t}=\frac{{I}_{CK{D}_{t}}}{{I}_{CS{I}_{t}}}\,\ast \,[Dose]$$

Here, all NM located within CKD and CSI compartments have both undergone an averaged media and cell-induced degradation. This calibrated fluorescence approach thus implicitly accounts for NM degradation without further data processing and requires no external calibration curve. For comparison, raw concentrations were obtained from the intensity of the CKD compartment at time *t* relative to the CSI compartment at time 0 (replace *I*_*CSI*,*t*_ with *I*_*CSI*,*0*_ in Eq. 1) to understand the degradative effect cells and media can have on a NM. Figure 2f shows results obtained from usage of Eq. (1) for calibrated and raw data calculations.

To ensure repeatability, the CF assay was completed approximately 2 months apart with a new batch of thawed cells for both NM types (n = 2), with reproducible results (Supplementary Figs 7 and 8).

### Comparing results from assay for QSH and PS

**Figure 3:**
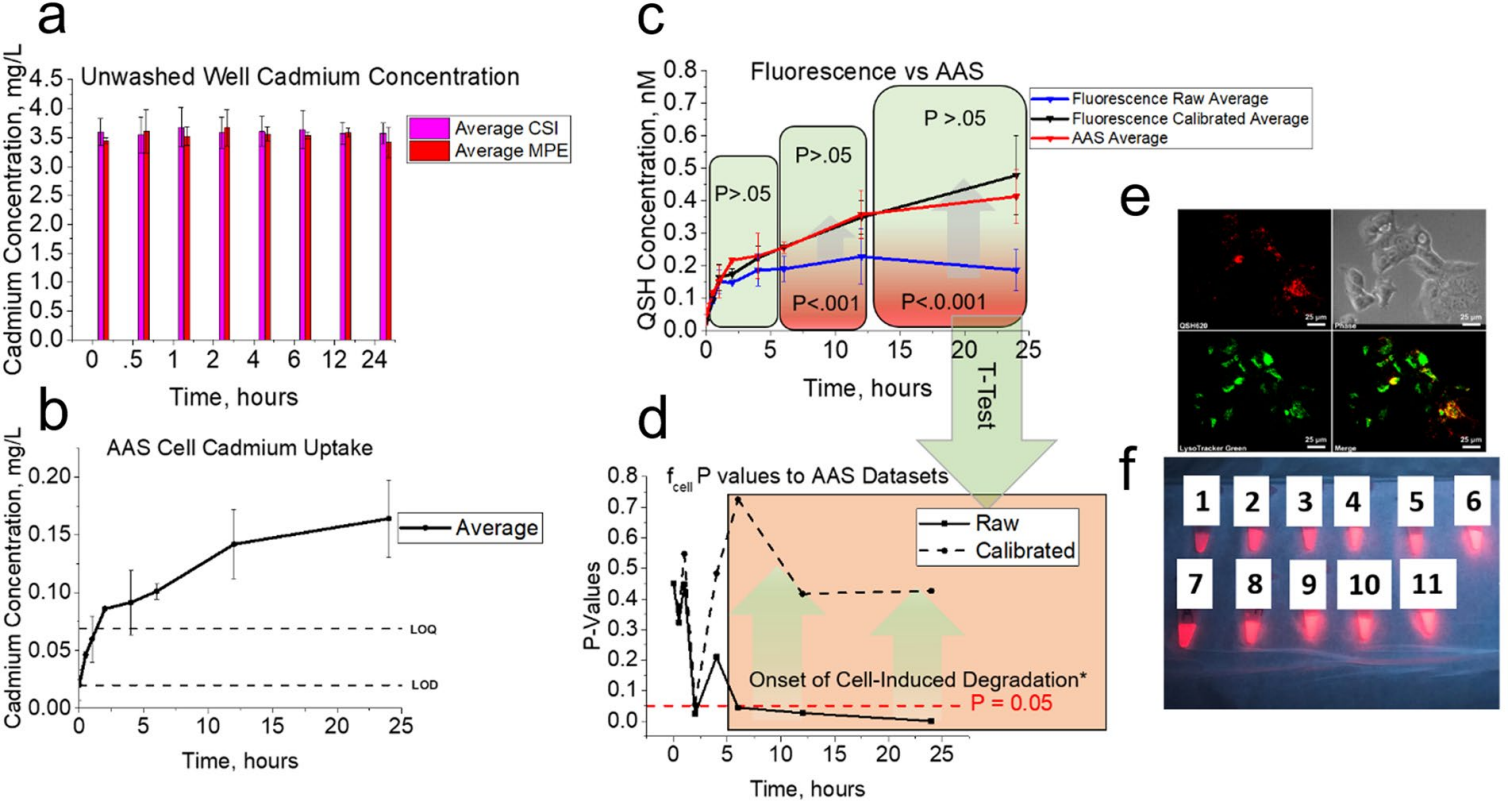
AAS analysis and validation to CF method. MPE and CSI compartments (**a**) contain 3.60 ± 0.0403 mg/L and 3.54 ± 0.0839 mg/L cadmium concentration, respectively. Data for CKD in (**b**) show gradual increase in cadmium concentration. Nanomolar quantities of QSH calculated from AAS and compared to fluorescence are shown in (**c**), with calibrated fluorescence results closely matching AAS average outputs. AAS average outputs were determined using a serial dilution of QSH with reference to a 6-point Cd calibration curve. (**d**) A two-tailed t-test p-value analysis between QSH concentrations adsorbed/internalized as determined by AAS, and calibrated (dotted line) or raw (solid line) data from *in vitro* assay accounting for or not accounting for cell-induced degradation, respectively, was performed. Nanomolar quantities shows increase in p-value when correcting for cell-induced degradation (calibrated). Asterisk after cell-induced degradation implies onset of significant increase in p-value when accounting for cell induced degradation. (**e**) shows lysosomal colocalization studies performed for QSH after cell exposure, with significant lysosomal sequestration. (**f**) gives visual evidence of fluorescence loss upon exposure to lysosomal conditions. After 24 hours, a snapshot was taken to illustrate the diminished fluorescence due to citric acid compared to HCl exposure. Solutions 1-6 were made with equal concentrations of 10 nM QSH with varying pHs (3.0–7.0, left to right) using differing concentrations of citric acid and sodium citrate (monobasic) buffer solutions made in distilled water. Solutions 1-6 contained pHs of 3.0, 3.5, 4.0, 4.5 5.0, and 7.0 (no citric acid or HCl i.e. water control), respectively. Solutions 7-11 contained HCl at pHs of 3.0, 3.5, 4.0, 4.5, and 5.0, respectively.

A key consideration to make was that data from the CSI compartment in Fig. 2a indicate media induced degradation from 30 minutes and cell induced degradation from 4 hours for QSH, while Fig. 2b shows no cell induced degradation and minimal media induced degradation occurring after 6 hours for PS. This was further tested by prolonged intracellular exposure studies, which resulted in a continued decrease in fluorescence signal (degradation) observed for QSH and full stability for PS (Supplementary Fig. 9). These results indicate that the collected fluorescence signal can be sensitive to the intracellular and media environment depending on the NM studied. All averaged cell- and media-induced degradation values (I_cdeg,*t*_ and I_mdeg,*t*_, respectively) are quantified in Fig. 2c. Evidence of cell-induced degradation is given by lysosomal colocalization (Fig. 3e) studies, observation of substantial Cd^2+^ core leakage (Supplementary Fig. 5) under simulated lysosomal conditions (Supplementary Fig. 10 and visual in Fig. 3f)^43,44^ and fluorescence data under cell exposure (Supplementary Fig. 11). More importantly, simulated lysosomal conditions (buffer vs non buffer) (Supplementary Fig. 11) indicate that the loss in QSH fluorescence and subsequent degradation is due to a contribution of the chelation effects from the citric acid as well as pH effects. Our *in vitro* studies with cells also showed minimal export of free Cd^2+^ from cells to the supernatant (Supplementary Fig. 12), not with a high enough sequestered concentration to exhibit a toxic response (Supplementary Fig. 13). Raw fluorescence data for QSH and PS collected from the CKD compartment (Fig. 2d,e) show an overall increase of raw fluorescence signal over time for both NMs, but with different profiles. The raw QSH profile rises to a maximum at approximately 12 hours, while the raw PS profile rises and saturates within 1 hour. Note that these raw fluorescence signals represent data that include degradation effects in the case of QSH.

Application of Eq. 1 ensures that degradation effects are accounted for during calculation of NM concentrations (refer to supplementary text for proof) since we compare I_CKD,*t*_ with I_CSI,*t*_ at their respective time points. By doing so, we internally calibrate the raw fluorescence signal collected from the (washed) CKD compartment to the (unwashed) CSI compartment (internal standard), knowing that both compartments degrade under the same conditions, and we obtain calibrated NM concentrations that are independent of NM degradation. Calibrated QSH concentrations (Fig. 2f) show a completely different profile from QSH concentrations calculated based on the corresponding raw fluorescence data (for calculation, see online methods). A non-saturable uptake is found for the calibrated QSH concentration as a function of time with C_max_ occurring at 24 hours (0.478 ± 0.122 nM), and significant deviation between calibrated and raw profiles is observed for QSH starting from approximately 4 hours, which is when cell-induced degradation begins (Fig. 2a). Raw fluorescence-based QSH concentrations (Fig. 2f) indicate saturable cell uptake, with maximum concentration (C_max_) occurring at approximately 12 hours (0.228 ± 0.0852 nM). In comparison, the calibrated and raw PS uptake profiles are not statistically different (P > 0.05), and reach saturability within 1 hour of exposure (Fig. 2f blue, solid and dashed). Overall, we found a fraction of the dose up to 4.78 ± 1.22% QSH and 1.07 ± 0.085% PS was adsorbed to/internalized by cells after 24 hours with respect to the initial applied dose.

### Atomic absorption spectroscopy analysis and validation of assay

AAS was run to validate the CF assay using absolute cadmium content with respect to QSH for the two studies (n = 2) discussed above. Samples were collected from all compartments in the CF assay at each time *t*, degraded in 33% v/v aqua regia (AR), and measured for absolute cadmium content. Note that AR treatment leads to a dilution of [Dose] from 10 nM to 6.66 nM that is accounted for in calculations (see online methods). Atomic absorbance measurements were referenced to a 6-point cadmium calibration curve (Supplementary Fig. 14) constructed with equal %v/v AR. As expected, AAS data obtained from the CSI and MPE compartments show that the cadmium concentration in both scenarios remained equal (P > 0.05) at constant concentrations of approximately 3.60 ± 0.0602 mg/L and 3.54 ± 0.0841 mg/L (Fig. 3a), respectively, since no cadmium is removed from the system for these samples (unwashed).

Parallel studies using a sample of QSH stock diluted to 6.66 nM showed no significant difference (P > 0.05, Supplementary Fig. 15), indicating quantitative collection of Cd^2+^ from the 96-well plates (CF assay). Extraction and harvest efficiencies for each time point were also determined to understand if the full dose of cadmium was extracted from the cells and harvested from the wells, with all results showing full extraction and harvest efficiency (Supplementary Figs 16-17).

AAS data obtained from the CKD compartments (Fig. 3b) show a gradual increase in total Cd^2+^ content, up to an average of 0.164 ± 0.0332 mg/L, which corresponds to 4.56 ± 0.925% of the applied dose. All experimental data lay significantly above both the limits of detection and quantitation (LOD and LOQ, respectively) with Pearson’s coefficient of 0.999 (Supplementary Table 3). Using these data, cadmium concentrations from AAS were converted to nM concentrations of QSH through a linear correlation of the slopes of the QSH and Cd(NO_3_)_2_ AAS calibration curves (online methods and Supplementary Fig. 18). We also performed a standard additions method and 6-point calibration method in parallel for the 24 hour time point (Supplementary Fig. 19a). Results did not differ significantly (P > 0.05, Supplementary Fig. 19b), indicative of minimal cell matrix interference on AAS data.

All data in Fig. 3c indicate similar QSH uptake up to 4 hours (P > 0.05, Fig. 3d) suggesting no significant degradation occurs. After 4 hours, as CSI and MPE intensities in Fig. 2a indicate, cell-induced degradation takes effect, and raw QSH concentrations obtained from the *in vitro* assay begin to saturate and deviate from AAS as well as from the CF-based QSH concentrations (Fig. 3c,d). AAS and CF-based QSH concentration uptake profiles are equivalent for all time points and do not differ with statistical significance. Profiles for the calibrated fluorescence concentrations match AAS, thus validating the fluorescence portion of the FORECAST method.

### Cell simulation quantifies kinetics and gives insight into NM localization on cell membrane and in cell space

A kinetics-based *in vitro* model was built to extract rate constants for adsorption, desorption, internalization, and degradation of NMs utilized in the CF assay. This cell kinetic model was guided by assumptions critical to biological processes involved in NM cell uptake including NM adhesion, desorption, internalization, and degradation through fist-order rate constants k_ads_, k_des_, k_int_, and k_deg_.

The model consists of compartments representative of (1) media, (2) cell membrane, and (3) cell space. These compartments are interconnected through basic mass transfer equations and first order rate constants. The cell kinetic model assumes a simple 3 compartment model, enough to provide sufficient understanding of whether a NM has made it inside the cell, remained outside, and how much it degraded.

#### Media compartment

This compartment includes the media environment from which cells receive their respective NM dose. The initial dose condition was taken as the 10 nM applied in the CF assay. The media compartment NM dose evolution with time is then described as:

2 $$\frac{d[Med]}{dt}=-\,{k}_{ads}\ast [Med]+{k}_{des}\ast [Mem]$$

where [Med] is concentration (nM) of NM in media, [Mem] is the concentration (nM) of NM adhered to cell membrane, and k_ads_, k_des_ are the first-order rate constants for adsorption and desorption to and from the cell membrane, respectively.

#### Cell membrane compartment

The cell membrane compartment is defined as the outer boundary of the cell with which the NM reversibly binds. This compartment separates the media from the internal space of the cell. NMs that are internalized by the cell must first adsorb to this compartment through the adsorption rate constant, k_ads_. Once adsorbed, NMs can (1) leave this compartment through desorption, k_des_ or (2) enter the cell via k_int_ as expressed by:

3 $$\frac{d[Mem]}{dt}={k}_{ads}\ast [Med]-{k}_{des}\ast [Mem]-{k}_{int}\ast [Mem]$$

with parameters described above.

#### Cell space compartment

The cell space compartment receives NMs that have transported inside the cell via the first order rate constant for internalization (k_int_). Here, NMs can become degraded if the process occurs (determined through CF assay). The cell space compartment NM dose evolution as a function of time is then described as:

4 $$\frac{d[Cell]}{dt}={k}_{int}\ast [Mem]-{k}_{deg}\ast [Cell]$$

where [Cell] is the concentration (nM) of QSH in cell interior at time t and k_deg_ is the first order rate constant for degradation of QSH obtained from optimization to raw datasets (see below).

**Figure 4:**
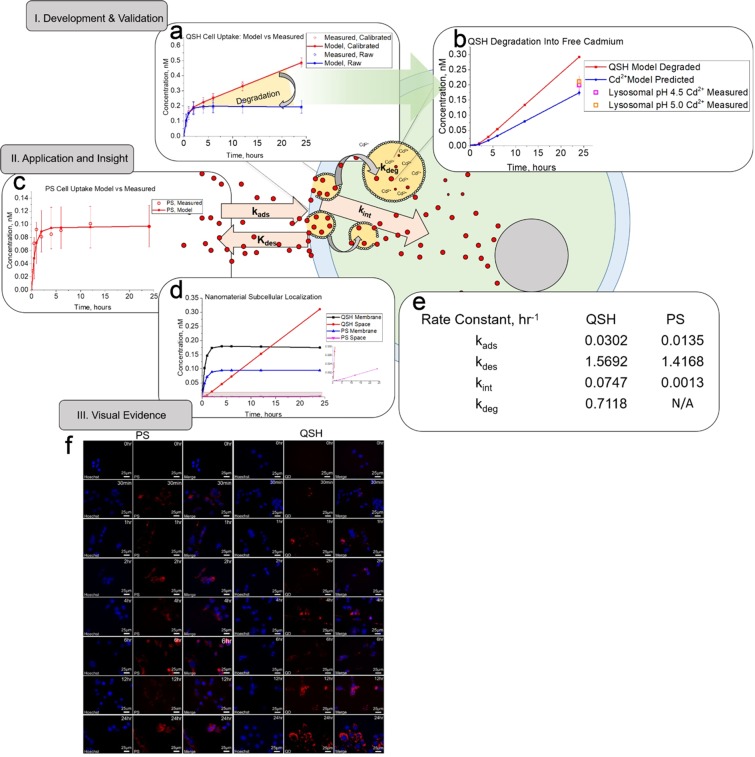
Outputs of cell simulation quantify rates of adsorption (k_ads_), desorption (k_des_), internalization (k_int_) and degradation (k_deg_) of NMs. (**a**) QSH model fits (line) to data (open circle) for cell samples taken from CKD for raw (blue) and calibrated (red) CF datasets. Error bars indicate 95% confidence for model fit to data. (**b**) Model output prediction for amount of QSH degraded (red) and subsequent free cadmium formation (blue) predicted by model. Experiments with simulated lysosomal buffer give comparable free cadmium release from degrading QSH (purple and orange squares). (**c**) Application of CF assay and cell simulation to stable PS nanoparticles shows early saturation compared to QSH QDs. (**d**) Cell simulation provides data on localization of NM on cell membrane or in cell space from CF datasets, showing membrane and internalized quantities. (**e**) Table summarizing kinetic rate constants obtained from FORECAST. Rate constants include adsorption (k_ads_), desorption (k_des_), internalization (k_int_), and degradation (k_deg_) for QSH QDs and PS NMs. (**f**) Confocal imaging of Hepa1-6 cells dosed with 10 nM QSH and PS. Cells were collected from CF assays. PS and QSH (red) images are shown above. Hoechst33258 (blue) is the nuclear stain. QSH shows no saturability for all time points from 0 to 24 hours. PS shows saturable uptake around 2 hours.

Equations 3–4 were first fit to calibrated CF data (QSH calibrated, PS, i.e. degradation effects removed) through optimization of parameters k_ads_, k_des,_ and k_int_. Here, we assume these data contain no degradation effects because the data were already calibrated to remove degradation effects through Eq. 1. Thus k_deg_ in Eq. 4 was held constant at 0 in this first optimization cycle for k_ads_, k_des,_ and k_int_. The genetic algorithm (GA)^36^ was chosen for optimization of the rate constants due to its usefulness in exploring a vast number space and application to machine learning techniques (e.g. smart nanoparticle design^45^). The GAs iterative approach, often times, leads to a global minimum^46^, and thus, has even been used to train artificial neural networks for drug design^47^. The GA is a robust artificial intelligence-based algorithm which undergoes optimization of parameters based on evolutionary ideas of natural selection. Initial vectors (“chromosomes”) comprised of rate constants (“genes”) were randomly populated by the GA, fed to the cell kinetic model, calculated for fitness, underwent selection, crossover, and mutations to maximize diversity and produce better fitness at each iteration (“generation”). At model convergence (20-50 generations, Supplementary Fig. 20), the optimized parameters provided reasonable correlation coefficients (R = 0.994 and 0.938 at P < 0.001), coefficients of determination (R^2^ = 0.989 and 0.880), standard errors (S = 0.0152 and 0.0158 nM), and residuals for QSH and PS respectively (Supplementary Table 4 and Supplementary Fig. 21). Next, raw concentrations (containing degradative effects) were used as the target for optimization of the degradation rate (k_deg_) by holding the previously optimized adsorption, desorption, and internalization rates (k_ads_, k_des,_ and k_int_) as constant. Thus we obtain k_deg_ from the difference between the calibrated and raw datasets. At model convergence (Supplementary Fig. 22), visuals of model outputs show reasonable fit to calibrated CF data for QSH and to raw QSH assay data with degradation (calibrated and raw datasets, respectively in Fig. 4a). All optimized rates were checked for global minimum of the GA through the following measures: (1) re-running the GA with increased mutation rates to maximize diversity (2) increasing generations (iterations) and (3) broadening the number space through increasing the population range to numbers that are significantly smaller and larger than what is experimentally appropriate based on CF data (e.g. a k_ads_ of 1 × 10^10^ hr^−1^ would suggest all of the dose will become membrane bound within a fraction of a second, which is not experimentally appropriate). Furthermore, as reported in literature^24,48–52^, NMs within this size range are primarily internalized via clathrin-mediated endocytosis, which is a time-driven process that requires (1) reversible binding to the cell membrane and formation of clathrin-coated pits (CCPs) and (2) internalization via clathrin coated vesicles (CCVs). This overall processes requires anywhere from 45 to 202 seconds to complete, and in many cases are mostly abortive (dissociative or cause desorption) for NMs within the vesicle size range^48,52^. This results in a balance between abortive events (k_des_), receptor binding and vesicle formation (k_ads_) and subsequent internalization(k_int_). The values we obtain from our simulation are reasonable, given this time-course of NM uptake. Interestingly, with the optimized rate of degradation k_deg_, the model predicted the concentration of QSH degraded (nM), and when converted to Cd^2+^ predicted absolute cadmium content that fits very closely to measured Cd^2+^ release from QSH under lysosomal conditions (Fig. 4b). This further illustrates our model’s predictive power to quantify NM degradation when used in conjunction with the CF assay.

For the PS NM, Fig. 4c shows the model fit to measured data. NM localization analysis (Fig. 4d) shows remarkable differences for both NM types. QSH membrane quantities were initially higher, until 12 hours, where internalized quantities dominated the total cell space. PS maintained high membrane binding with minimal internalization for all time points. Rate constants (Fig. 4e) indicate that membrane binding is the rate limiting step for QSH, while internalization is the rate limiting step for PS. This is in agreement with literature, as membrane binding (clathrin-coat ligand bonding and formation) are often the rate limiting steps^50^. Confocal microscopy (Fig. 4f) qualitatively supports these findings, with saturable uptake for PS within 2 hours and limited saturability for QSH with higher internalization.

## Discussion

FORECAST introduces a new tool to quantify NMs’ mechanistic biological properties by providing a predictive means of understanding kinetics of NM-cell interactions and NM localization on the cell membrane or in the cell space. We have developed FORECAST as a quantitative coupled *in vitro* experiment-simulation method to determine the kinetics of NM interaction with cells. In our approach we utilize an artificial intelligence algorithm to extract quantitative kinetics from an *in vitro* assay. These outputs can be coupled to *in vivo* whole-body animal simulations. CF data for FORECAST was validated extensively through AAS analysis. Our method is high throughput and allows for efficient screening to collect data on libraries of NMs.

We envision this technique being used in a variety of labs to capture and understand accurate NM localization on the cell membrane or in cell space with newly-developed NMs, because of the ease of use of the method, its high throughput screening capability, and the quantitative biologically relevant parameters that are obtained. The method also provides a pathway to predicting *in vivo* biodistribution that may eventually reduce the need for animal testing.

### Advantages and limitations of the FORECAST method

Our method makes use of the combined *in vitro* assay-simulation approach in a way that is simple to apply in the lab. Labs that have cell culture capability and access to a plate reader and computer can use the reported method. The major disadvantage of our method is that NMs have to be fluorescent. NMs can be made fluorescent with dye coating but it could lead to questions about changing the nature of the NM surface, and would require developing proof, if possible, that there is no difference. Since the *in vitro* assay delivers concentrations of NM for the intracellular and extracellular compartments, other analysis methods such as e.g. ICP/MS or GC/MS could determine these data for non-fluorescent NM. The cell kinetics model would then still apply without further modification. Samples that show full loss of fluorescence on the time scale of the assay would not work, although the time of the assay could be shortened to accommodate.

When NMs exhibit sedimentation or buoyancy it may affect the *in vitro* assay results. In these cases, the exposure dose to cells would be variable, increasing or decreasing over time, and deviate from the applied (nominal) dose. Thus, measured NM uptake by cells would be greater or less than expected (given a nominal dose) if sedimentation or buoyancy is present, respectively. However, it is likely that, given the physical properties of the particles used in this study, sedimentation or buoyancy is not a significant factor (also refer to Supplementary Fig. 3). The QSH QDs are near the 10 nm size for which sedimentation may be minimal and convective forces are the primary driving force of exposure to cells^42^, while the PS particles used in our studies are larger but of lower density. For studies with particles that do exhibit sedimentation or buoyancy, the use of transport simulation models such as ISDD (*In vitro* Sedimentation, Diffusion and Dosimetry)^53^, ISD3 (*In vitro* Sedimentation, Diffusion, Dosimetry and Dissolution)^54^, or DG (Distorted Grid)^55–57^ assists in predicting particle dose at the cell surface over time relative to the starting concentration. In such cases, FORECAST would need to be used in coordination with, or ideally integrate one of these transport simulation models. Specifically, Eq. (2) media concentration ([Med]) would receive input from the sedimentation models, and thus [Med] would no longer represent the nominal (applied) NM dose but the effective exposure dose at the cell surface, which can then change over time. The integration of FORECAST and transport simulations would thus provide the necessary adjustments for a more accurate cell dosimetry model needed for extrapolation to PBPK simulations.

### Importance of the method

NM kinetic data representing interaction with cells is required for accurate predictive modeling of NM *in vivo*, but has been challenging to deliver to the pharmacokinetic modeling field. With our method, these data can be obtained quantitatively. Rate constants optimized to cell uptake data, obtained from our method, are valuable in that they may couple directly to *in vivo* biodistribution modeling. This enables predictive power for NM animal simulations while obtaining biodistribution data with insight onto NM accumulation on the cell membrane and cell space starting from *in vitro* assay alone. Tissue level resolution can now be enhanced to the cellular level through rate constants determined from the *in vitro* portion of the FORECAST method. In the future, this method can provide a streamlined approach to enable much quicker and cost-effective NM screening for nanomedicine, public safety, and answering broader regulatory questions.

## Methods

### Materials

Commercially available quantum dot QSH620 (negatively charged CdSe/ZnS core-shell), and carboxy-functionalized polystyrene nanoparticles (PS, CAF-050NM red fluorescent) were purchased from Ocean NanoTech, LLC and Magsphere, Inc, respectively. All materials were used as-is, unless otherwise specified.

### Physicochemical characterization

The size of QSH620 and PS was obtained with a Malvern Zetasizer. Samples were diluted in nanopure water at concentrations of 10 nM and measured immediately. Zeta potential measurements were performed similarly. Absorption spectra were obtained using an Agilent 8543 UV-Vis spectrophotometer. Fluorescence measurements were obtained with a top-read Tecan M200 Pro plate reader with excitation at approximately 580 nm for QSH and 525 nm for PS.

### *In vitro* statistical analysis

Statistical analysis was performed on Microsoft Excel 2010. All calculated statistical evaluations were performed using the student’s two-tailed t-test at the P < 0.05, P < 0.01, or P < 0.001 level.

### Processing and reading *In vitro* assay in CF approach

All fluorescence measurements were taken with a top-read Tecan M200 Pro Plate Reader. Fluorescence for QSH was taken with excitation of 580 nm and emission of 620 nm. PS was excited at 525 nm and emission was collected at 595 nm. All plate reader measurements were performed using four reads per well to obtain statistically relevant data. For compartments CSI, CKD, and controls, 100uL of a 347,000 cells/mL cell suspension (Hepa1-6, murine hepatoma cell line from ATCC) was applied and incubated for 48 hours in a black 96 well plate (96 well solid black flat bottom polystyrene TC-treated microplates, Corning) to allow for 80-90% confluence. At time of experiment, all compartments were washed 1X with complete growth media, aspirated, and 100uL aliquots of 10 nM QSH or PS concentrations in DMEM (DMEM/Ham’s F-12 50/50 Mix, Corning) supplemented with 10%FBS (Regular heat-inactivated, Corning) was added to all compartments (CSI, MPE, and CKD) in plates designated for that particular NM type. For QSH, each well plate consisted of 18 replicates of each compartment, specifically CSI, MPE, CKD, and controls (CC) for 0, 0.5, 1, 2, 4, 6, 12, and 24 hours. For PS, one triplicate for MPE, CSI, and control was used, *but with 1 triplicate designated for each time point for CKD*. In both scenarios for NMs, at each time point, CKD wells were washed 2X with complete growth medium, 1X with PBS (Phosphate buffered saline, ThermoFisher Scientific), and trypsinized for 15 minutes (0.25% in EDTA, Invitrogen), mixed, and *fluorescence plate readings run on all well types (CSI*, *MPE*, *CKD*, *and control) with an excitation of 525 or 580 and emission at 595 or 620* *nm (for QSH or PS*, *respectively)*. All fluorescence values were corrected for media and trypsin background signal. Specifically, I_CSIt_, I_MPEt_, I_CKDt_, were all read at these time points. I_cdeg,t_ and I_mdeg,t_ were taken as the difference of I_MPEt_ and I_CSIt_ as well as I_MPE,0_ and I_MPEt_, respectively. Note: 0 hours consisted of an entire 96-well plate with aspiration immediately (<2 minutes) after application to all wells and subsequent washing and trypsinization of CKD. Fluorescence plate readings took approximately 2-5 minutes per plate.

### Cf parameter outputs and usage

Signals obtained from CF assay include:

1. *I*_*CSI*,*0*_
2. *I*_*CSI*,*t*_
3. *I*_*MPE*,*0*_
4. *I*_*MPE*,*t*_
5. *I*_*CKDt*_
6. *I*_*CC*_

Overall, raw fluorescence descriptive of cell uptake (*I*_*CKD*,*t*_) was taken relative to raw fluorescence of unwashed cells at time t (*I*_*CSI*,*t*_) to obtain a calibrated fraction of uptake (*f*_*cell*,*c*_):

5 $${f}_{cell,c}=\frac{{I}_{CK{D}_{t}}}{{I}_{CS{I}_{t}}}$$

This fraction of uptake is plotted in Supplementary Figures 7 and 8 for both NM types. Raw fluorescence descriptive of cell uptake (*I*_*CKD*,*t*_) was also taken relative to raw fluorescence of unwashed cells at time 0 (I_CSI,0_) to obtain a raw fraction of uptake (*f*_*cell*,*r*_):

6 $$fs=\frac{{I}_{CK{D}_{t}}}{{I}_{CS{I}_{0}}}$$

These two fractions were then used to obtain concentration of NM uptake using the general equation,

7 $${[Uptake]}_{c,t}={f}_{cell,x}\ast [Dose]$$

where *f*_*cell*,*x*_ is the fraction of uptake for x = raw or corrected, *[Uptake]*_*t*_ is the concentration of NM taken up by cells (nM), and *[Dose]* is the applied dose in nM. To determine if cell-induced degradation is present, a two-tailed t-test was performed between unwashed CSI and MPE compartments at time, *t*, (${I}_{CS{I}_{t}}\,and\,{I}_{MP{E}_{t}},\,respectively$). To determine if media-induced degradation is present, a two-tailed t-test was performed between unwashed MPE at time 0 and time *t*.

Cell-induced degradation $({I}_{cde{g}_{t}})$ was taken as the difference between unwashed without $({I}_{MP{E}_{t}})$ and with $({I}_{CS{I}_{t}})$ cell exposure,

8 $${I}_{cde{g}_{t}}={I}_{MP{E}_{t}}-{I}_{CS{I}_{t}}$$

If media degradation was present, the intensity of this degradation type was taken as the difference between unwashed wells without cell exposure from time 0 to time t.

9 $${I}_{mde{g}_{t}}={I}_{MP{E}_{0}}-{I}_{MP{E}_{t}}$$

Taken together, the sum of these values equals the total degradation that a NM can undergo for the CF assay $({I}_{de{g}_{t}})$:

10 $${I}_{de{g}_{t}}={I}_{cde{g}_{t}}+{I}_{mde{g}_{t}}$$

### AAS sample preparation

For AAS validation of the above fluorescence CF results, 50uL aliquots of aqua regia was added to every experimental well (both to unwashed/washed and cells/no cells) and left to digest for 10 minutes in incubator. In this case, every well received equal dilution for proper calibrated quantitation. At time, homogenized solutions were collected and transferred to sealed glass vials for atomic absorption experiments. Care was made to ensure wells were kept at equal dilutions, 150uL of total solvent per well, *leading to a theoretical QSH concentration of 6*.*66* *nM for all unwashed wells*. Wells with unknown concentrations (washed cells) were diluted equally as wells with known concentrations of QSH. A Perkin Elmer Analyst 400 AAS spectrometer was used to analyze cadmium solutions. A cadmium hollow cathode lamp with wavelength of 288.65 nm was used to obtain optimal cadmium absorption. Flow rate was adjusted to 4 mL/min and samples were run in triplicate. A 6-point standard calibration curve was constructed with NIST grade Cd(NO_3_)_2_ in AR. Care was taken to ensure that sample readings were within the linear dynamic range of the calibration curve and limit of quantitation (LOQ) of the instrument.

### AAS qsh concentration determination from Cd

First, mirroring the calibrated assay design, AAS cadmium concentrations from CKD were divided by total unwashed CSI cadmium concentrations at time *t* and multiplied by the QSH dose applied to cells (in nM) to yield a nM concentration of QSH (Supplementary Fig. 23, Calibrated), according to equation

11 $${[Uptake]}_{c,t}=\frac{{[Cd]}_{CK{D}_{t}}}{{[Cd]}_{CS{I}_{t}}}\ast [Dose]$$

where [Uptake]_c,t_ is the concentration of QSH taken bup by cells in nM, ${[Cd]}_{CK{D}_{t}}$ is the washed CKD compartment at time t containing cadmium concentration in mg/L obtained from AAS, ${[Cd]}_{CS{I}_{t}}$ is the unwashed total compartment containing cadmium concentrations in mg/L, and [Dose] represents total dose expressed in nM.

The second approach (Supplementary Fig. 23, Linear Correlation) was determined from the utilization of a standardization curve containing QSH concentrations of 0.25, 0.50, 1.00, 5.00, 10.00nM, and Cd(NO_3_)_2_ concentrations of 0.25, 0.50, 0.75, 1.00, 2.00, and 4.00mg/L. The slope of the Cd(NO_3_)_2_ curve was then used to build a new curve correlating QSH nM concentrations to Cd mg/L concentrations. These values were then used to compare AAS results to those in the CF method.

### Assessment of NM toxicity

MTS (CellTiter 96 AQ Non-Radioactive Cell Proliferation, VWR) assay was run to determine toxicity of a variety of NMs at different doses (QSH and PS) for optimal NM exposure conditions. NMs were applied to murine Hepa1-6 cells for a period of 24 hours. Briefly, cells were seeded in triplicate onto wells of a clear flat bottom 96-well plates at a density of 34,700 cells/well and left 24 hours for attachment. At time, media was aspirated, and 100uL of all NM solutions were applied to wells, except controls, for a period of 24 hours in 37 °C CO_2_ incubator. Negative controls were kept in media to retain complete viability and positive controls were kept in water for cell death. All NMs were diluted in DMEM supplemented with 10%FBS at various doses, ranging from 5nM to approximately 250nM. At time, cells were washed 2X with complete growth medium and re-applied with 100uL of DMEM with 10%FBS. 20uL aliquots of MTS was added and background absorbance was captured at 490nm. Plates were then incubated for 2 hours and absorbance checked again. Sample absorption values were normalized to that of cells exposed to complete growth medium.

### Assessment of QSH and PS stability

To assess what dose of QSH and PS would be optimal for the cell kinetic experiment media stability was determined. Time-dependent experimental data was taken for QSH and PS at various concentrations. QSH and PS solutions were made in DMEM supplemented with 10% FBS at concentrations 2.5, 10, 50, 100, 250, and 300nM. The solutions were applied in 100uL aliquots in triplicate to wells of a 96 well plate and left inside incubator for up to 24 hours. At each time point, separate pre-determined “mixed” wells were mixed and all wells (including mixed and non mixed) were measured for fluorescence with a constant z-optimized focal plane of 18181um, the maximum fluorescence signal-noise ratio. This value was kept constant throughout the time-dependent study to understand sedimentation effects. Mixed wells were included to insure there was no sedimentation occurring, as any difference between mixed and non mixed is indicative of sedimentation effects.

### Lysosomal colocalization studies

Cells were seeded onto 35mm diameter tissue coated petri dishes (35mm TC-treated culture dish, Corning) with 2mL of 347,000 cells/mL solution and left in incubator at 37 °C and 5%CO_2_ for 24 hours. Cells were washed 1X with complete growth medium and 2mL of 10nM QSH solutions were added. After 24 hours, petri dishes were removed from incubator and washed 2X with complete growth media. Lysotracker Green (DND-26, ThermoFisher Scientific) was added at 1uM concentration and confocal images obtained. Lysosomal colocalization studies were performed using a spinning-disk confocal imaging system. Z-stacks were taken at 2um step sizes, with a total distance of 40 um.

### Cell kinetic confocal microscopy

A paralleled series of CF cell kinetic samples containing either QSH or PS NMs were analyzed for uptake using confocal microscopy. At each time point in the CF study, cells were washed 2X, trypsinized, and transferred to 35mm petri dishes containing 2mL of complete growth medium. After 24 hours, cells were washed 2X with complete growth medium, and Hoechst33257 was applied. Cells were then imaged for NM uptake with 20 2 um step sizes.

### Simulated lysosomal buffer studies

Cellular lysosomal environment was mimicked to determine stress induced on fluorescence through lysosomal material exposure. The citric acid (, >99.5%, ACS Reagent, Sigma-Aldrich) simulated lysosome chelator buffer at pH 3.0-6.0 was created and used as the solvent for QSH and PS. Controls contained pH 7 nano-pure water solutions. More specifically, varying oncentrations of citric acid and sodium citrate (Anhydrous, Sigma-Aldrich, monobasic) were combined to acquire the desired pH. In parallel, equal concentrations of QSH and PS were created in HCl, with differing concentrations of HCl to achieve the desired pH. For size analysis, Zetasizer (Malvern) DLS measurements were obtained. Here, QSH and PS samples were diluted *in-situ* in solvents of desired pH and measurements obtained immediately after (for pH below 3.0, HCl was added). Fluorescence plate readings were run in triplicates of 100uL of solutions applied to wells of a 96-well plate system. Fluorescence was taken with 580 or 525 excitation and 595 or 620nm emission, respectively for QSH or PS, using a Tecan M200 plate reader.

To check for Cd^2+^ core leakage, 10nM QSH and PS were analyzed for fluorescence in PBS, water, and simulated lysosomal buffer at pH 2.5, 4.5, and 5.0 at 0 hours and 24 hours exposure. For each time point, samples were collected and centrifuged at 15,000xg for 20 minutes through an Amicon Ultra 10kDa filter to separate possible cations from QSH. Filtrate was then analyzed for free cadmium content using a PerkinElmer atomic absorption spectrometer with a cadmium hollow cathode lamp with wavelength of 288.65nm. Flow rate was adjusted to 4mL/min and samples were run in triplicate.

### Prolonged cell exposure study

Prolonged exposure to intracellular environment analysis was performed after washing at time, t. Here, QSH or PS washed samples at time, t, were left to incubate to an additional 12-x and 24-x hours, where x is the time of wash for each particular sample. At total experimental time of 12 and 24 hours, previously washed plates were mixed and measured for fluorescence changes from their original time, t. An example is shown below:

*2 hours* wash fluorescence → *10 hour post wash* (12-2 hours) fluorescence → *22 hours post wash* (24-2 hours) fluorescence

Importantly, 12 hour washed sample only contained 24-x prolonged cell exposure data and 24 hour washed sample contains no prolonged exposure, given that cell exposure was only allowed for the duration of a total time of 24 hours.

### Calculation of fluorescence plate reader limit of detection and quantitation

The limits of detection (LOD) and limits of quantitation (LOQ) were calculated from construction of an 8-point calibration curve with concentration ranging from 0.10-10 or 15nM for QSH or PS. The LOD and LOQ were calculated based on the standard deviation of the response signal of the blank and slope of the linear curve through zero, equations below:

12 $$\mathrm{LOD}\,=\frac{3{\rm{\sigma }}}{S}$$

13 $$\mathrm{LOQ}\,=\frac{\mathrm{10}{\rm{\sigma }}}{S}$$

where $\sigma$ is the standard deviation of the blank (NM suspension in trypsin) and S is the slope of the calibration curve. All readings were performed on a TecanM200 Pro.

### Perkin-Elmer aas limit of detection and limit of quantitation

The Perkin-Elmer AAS limits of detection (LOD) and limits of quantitation (LOQ) were calculated from construction of a 6-point calibration curve with concentration ranging from .25-4 mg/L. Media signal in 33% v/v AR was used as blank for these values. The LOD and LOQ were calculated based on the standard deviation of the response signal of the blank and slope of the linear curve through zero, see Eqs (8) and (9).

### Analysis of qsh cadmium core leakage in cell supernatant

Cells were plated onto wells of a 96-well plate system at 34,700 cells/well and left to incubate for 48 hours. At time, media was aspirated and 100uL aliquots of 10 nM QSH was applied. Cells were then placed in incubator for maximum experimental exposure time for 24 hours. After 24 hours, fluorescence measurements were taken for QSH with no cell exposure (I_MPE,t_) and with cell exposure (I_CSI,t_) to show difference in total fluorescence. All sample supernatant was collected separately and transferred to 2 mL centrifuge tubes with Amicon Ultra 10 K filters. Samples were centrifuged according to protocol at 14,000xg for 20 minutes to filter out solvent and possible free cadmium. Filtrate and concentrate were then collected analyzed on AAS for cadmium concentration.

### Extraction, collection, and harvest efficiencies

Extraction, collection, and harvest efficiencies were performed to ensure accurate and precise experimental protocol. For extraction efficiency, cell kinetic samples were washed 2X with complete growth media, trypsinized, digested *in-situ* and collected for AAS. In parallel, cells were washed 2X with complete growth media, not trypsinized, digested with AR *in-situ*, and collected for AAS. If full extraction of cadmium from cell interior is happening, both sample sets are expected to yield similar results, or close to 1 when normalized. Harvest efficiency was performed by collection of QSH from wells with no cells and wells with cells. In both cases, AR was applied *in-situ* and samples were collected for AAS. If full QSH cadmium harvest is apparent, then normalized data should yield values close to 1. Collection efficiency was performed by comparison of direct dilution of a separate vial of 10 nM QSH to 6.66 nM by AR application in parallel to QSH in wells with no cells and equal digestion *in-situ* with aqua regia. If all sample is collected from the wells, then the *in-situ* AAS values should yield similar results to the diluted vial and normalized close to 1.

### *In vitro* model global optimization setup and evaluation

All simulations were performed in MATLAB v2015b. Parameter optimization was implemented with the genetic algorithm (GA) optimization function from the Optimization Toolbox. Parameters for estimation included:

- *Initial Population*: 300
- *Population Size*: 50
- *Generations*: 100
- *Mutation Rate*: Mutation Gaussian
- *Crossover Rate*: .80
- *Selection Function*: Stochastic Uniform

The genetic algorithm was evaluated using the residual sum squares as the fitness function, equation below:

14 $$RSS=\mathop{\sum }\limits_{i}^{n}{({y}_{i}-{m}_{i})}^{2}$$

where RSS represents the residual sum of squares from model output (m_i_) at time (i) to measured data (y_i_) for n time points. Standard error was computed as

15 $$S=\,\sqrt{\frac{RSS}{n}}$$

where S is standard error, RSS residual sum of squares, and n is total time points.

Model output upper and lower bounds were evaluated at the 95% confidence interval through

16 $$CL(95 \% )=Model\,Output\pm 2\ast S$$

where CL(95%) represents 95% confidence limit.

The GA was run for 100 generations, enough to allow for convergence at a fitness value representative of measured data.

## Supplementary information

Supplementary Info
