# Author Correction: An *in vitro* assay and artificial intelligence approach to determine rate constants of nanomaterial-cell interactions

**DOI:** 10.1038/s41598-019-56347-5

**Published:** 2019-12-20

**Authors:** Edward Price, Andre J. Gesquiere

**Affiliations:** 1 NanoScience Technology Center, University of Central Florida, Orlando, FL 32826 USA; 2 Department of Chemistry, University of Central Florida, Orlando, FL 32816 USA; 3 Department of Materials Science and Engineering, University of Central Florida, Orlando, FL 32816 USA; 4 The College of Optics and Photonics (CREOL), University of Central Florida, Orlando, FL 32816 USA

Correction to: *Scientific Reports* 10.1038/s41598-019-50208-x, published online 26 September 2019

The Acknowledgements section in this Article is incomplete.

“We thank Michael Miller at W.S.I. for discussion.”

should read:

“We thank Michael Miller at W.S.I. for discussion. We gratefully acknowledge Dr. Tyler Maxwell for his contribution to Figure S11c.”

