# Supplementary material for: An in vitro assay and artificial intelligence approach to determine rate constants of nanomaterial-cell interactions
Source: Sci Rep. 2019 Sep 26;9:13943. doi: 10.1038/s41598-019-50208-x (PMC6763461; doi:10.1038/s41598-019-50208-x)
Supplement: Supplementary file 1 — Supplementary Info [file 41598_2019_50208_MOESM1_ESM.pdf]

Supplementary Information

An *in vitro* assay and artificial intelligence approach to determine rate constants of nanomaterial-cell interactions

Edward Price[1,2], Andre J. Gesquiere[*,1,2,3,4]

[1]NanoScience Technology Center, University of Central Florida, Orlando, FL 32826, USA

[2]Department of Chemistry, University of Central Florida, Orlando, FL 32816, USA

[3]Department of Materials Science and Engineering, University of Central Florida, Orlando, FL 32816, USA

[4]The College of Optics and Photonics (CREOL), University of Central Florida, Orlando, FL 32816, USA

[*] To whom correspondence should be addressed. E-mail: andre@ ucf.edu (A. J. Gesquiere)

Supplementary Tables

Table 1. Quantum Dot and Polystyrene Physicochemical Characterization

NMs	Size, nm[a]	Surface Chemistry	ζ Potential, mV[b]	Absorption, nm[c]	Fluorescence, nm[d]
QSH	13.6 ± 1.11	-COOH	-20.8 ± 9.77	580	620
PS	35.7 ± 6.15	-COOH	-25.9 ± 7.64	525	590

a Hydrodynamic diameter measured by DLS
b Zeta potential measured by zetasizer
c Absorption wavelength used in CF assay, determined through spectral analysis
d Fluorescence wavelength used in CF assay, determined through spectral analysis

Table 2. Quantum Dot and Polystyrene Limits of Detection and Quantitation

Sample ID	LOD, nM	StDev	LOQ, nM	StDev
QSH	0.004833968	3.54583E-05	0.016113226	0.000118194
PS	0.007989205	3.97261E-05	0.026630684	0.00013242

Table 3. LOD and LOQ Values for PerkinElmer Flame AAS

Cadmium LOD and LOQ Values ± Standard Deviation	
LOD (mg/L)	0.0207 ± 0.000284
LOQ (mg/L)	0.0691 ± 0.000950

Table 4. Model Output Statistics to Measured Datasets

Simulation Type	R	P-value	R Square	Std. Error, nM	Residual Sum of Squares, nM
QSH Model Calibrated	0.9944	0.0000	0.9889	0.0152	0.0019
PS Model	0.9380	0.0006	0.8798	0.0158	0.0020
QSH Model Raw	0.9471	0.0004	0.8969	0.0212	0.0036

Supplementary Figures

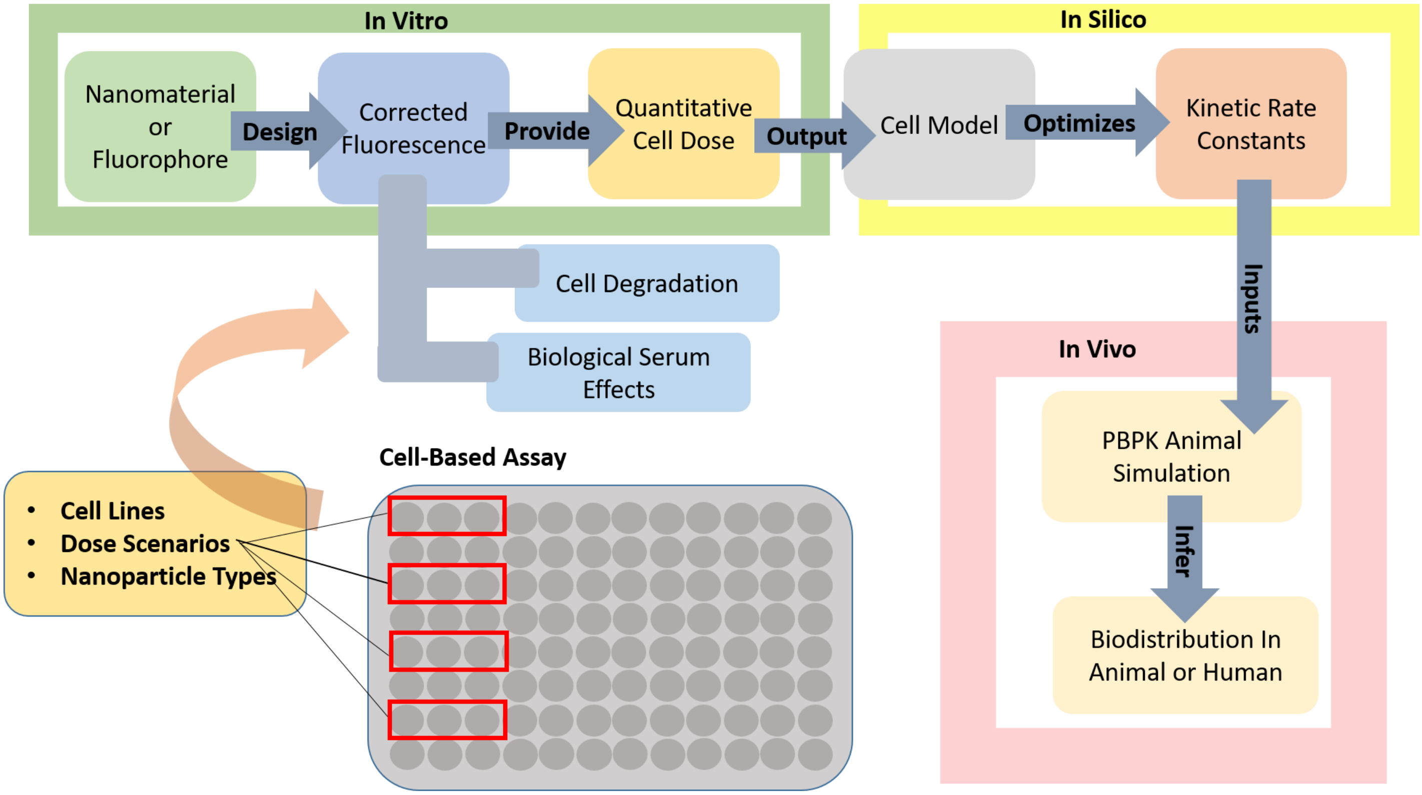

Supplementary Figure 1. Schematic representative of FORECAST and its potential application in predictive animal simulations. NMs or fluorophores must be chosen in order to run a fluorescent study on a variety of cell lines with different dosing scenarios on the cell-based assay. The cell based assay captures biological serum and cellular-induced degradative effects. Data obtained from the cell based assay provides a quantitative cellular dose that feeds to an in silico model. This model then optimizes rate constants, which serve as inputs towards in vivo PBPK models to capture accurate animal biodistribtuion.

b

a

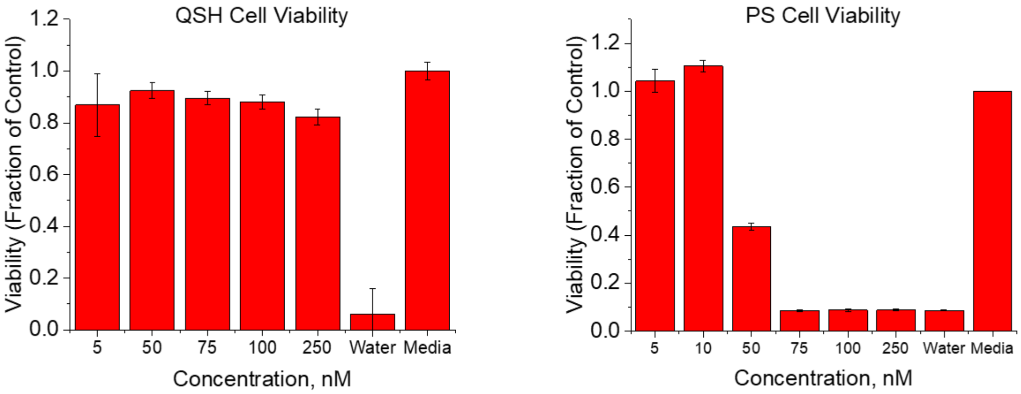

Supplementary Figure 2. MTS assay data for all NMs included in cell kinetic analysis. (**a**) QSH (negatively charged) and (**b**) PS. Negatively charged QSH experienced minimal toxicity for all doses, and PS exhibited minimal toxicity at 10nM or below. Positive control contained cells exposed to water and negative control contained cells exposed to complete growth media.

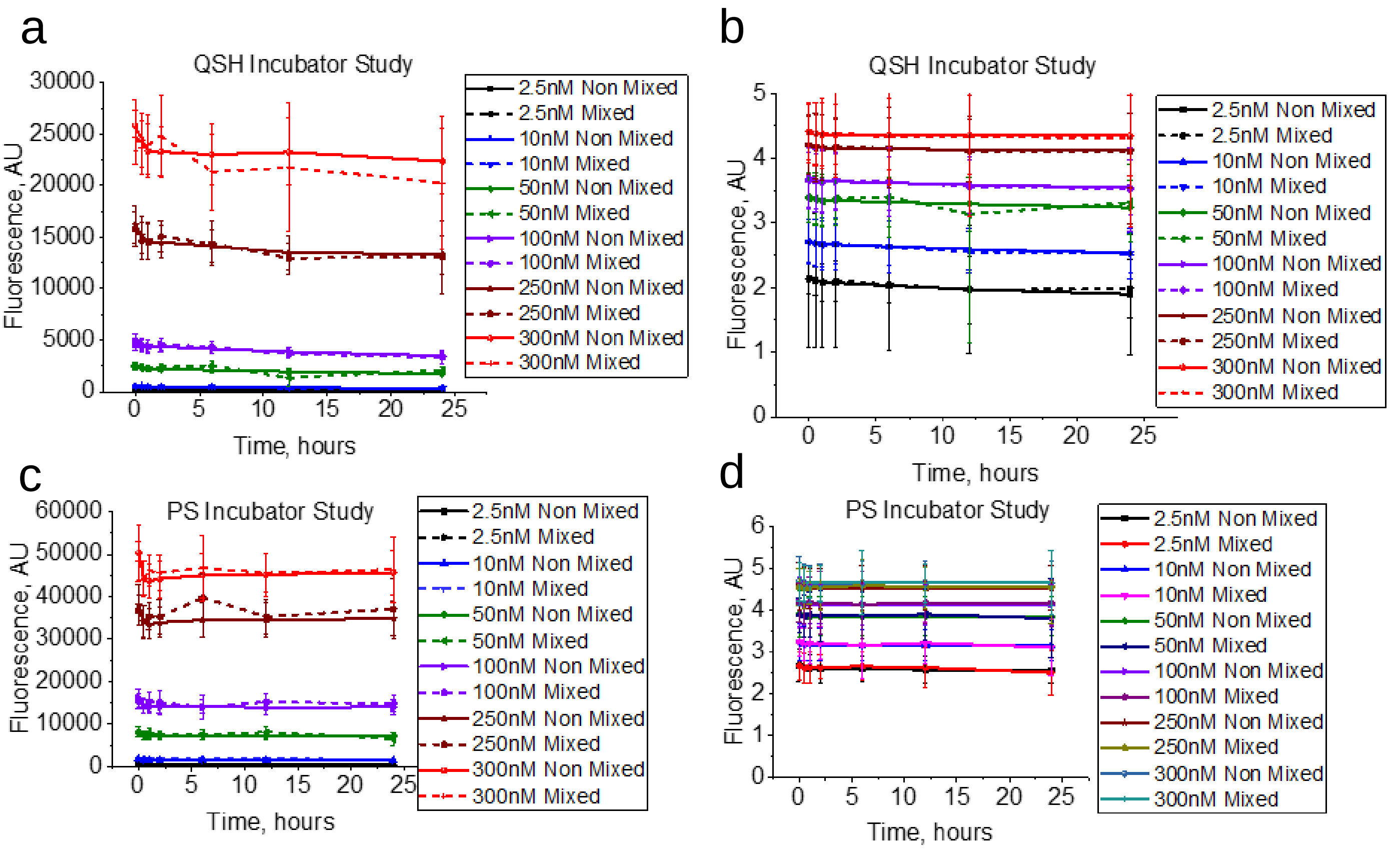

Supplementary Figure 3. QSH stability studies inside incubator at 37°C and 5%CO_2. Fluorescence results for (**a, b**) QSH and (**c, d**) PS indicate relative stability for concentrations used within the 2.5-300nM. Therefore, concentrations of 10nM for both NM types is reasonable enough for selection for cell kinetic studies. Optimized detector Z-plane was kept constant on the plate reader.

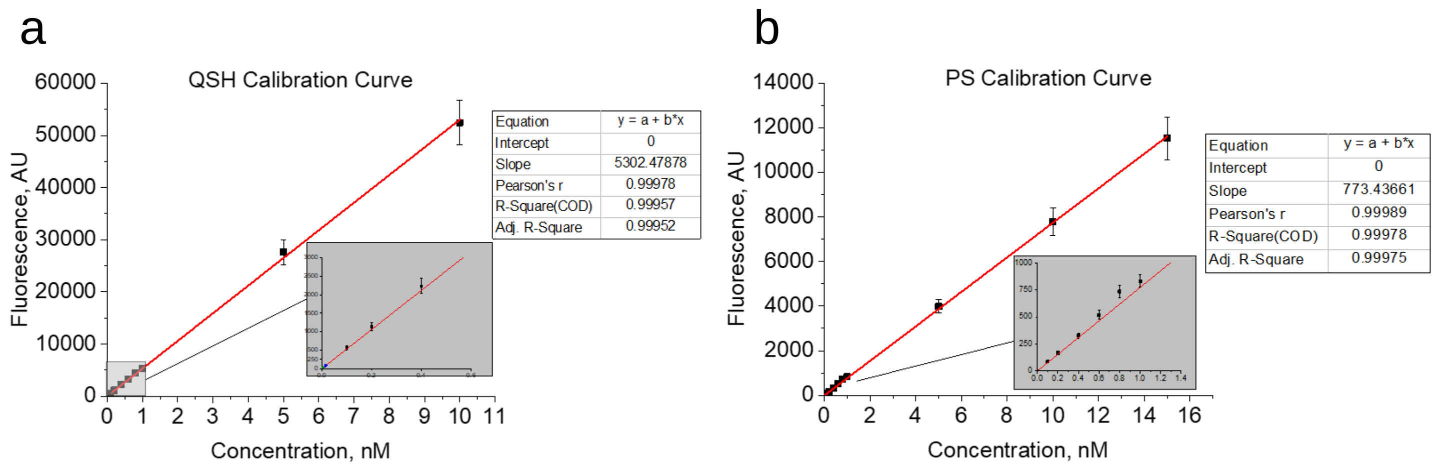

Supplementary Figure 4. QSH and PS calibration curves. An 8 point calibration curve was constructed with concentrations of .10, .20, .40, .60, .80, 1, 5, 10, and/or 15nM in complete growth media. The linear dynamic range (LDR, linearity) was noted for concentrations as low as .10nM and as high as 10 or 15nM for QSH and PS. These calibration curves were used to calculate limits of detection and quantitation for both NM types.

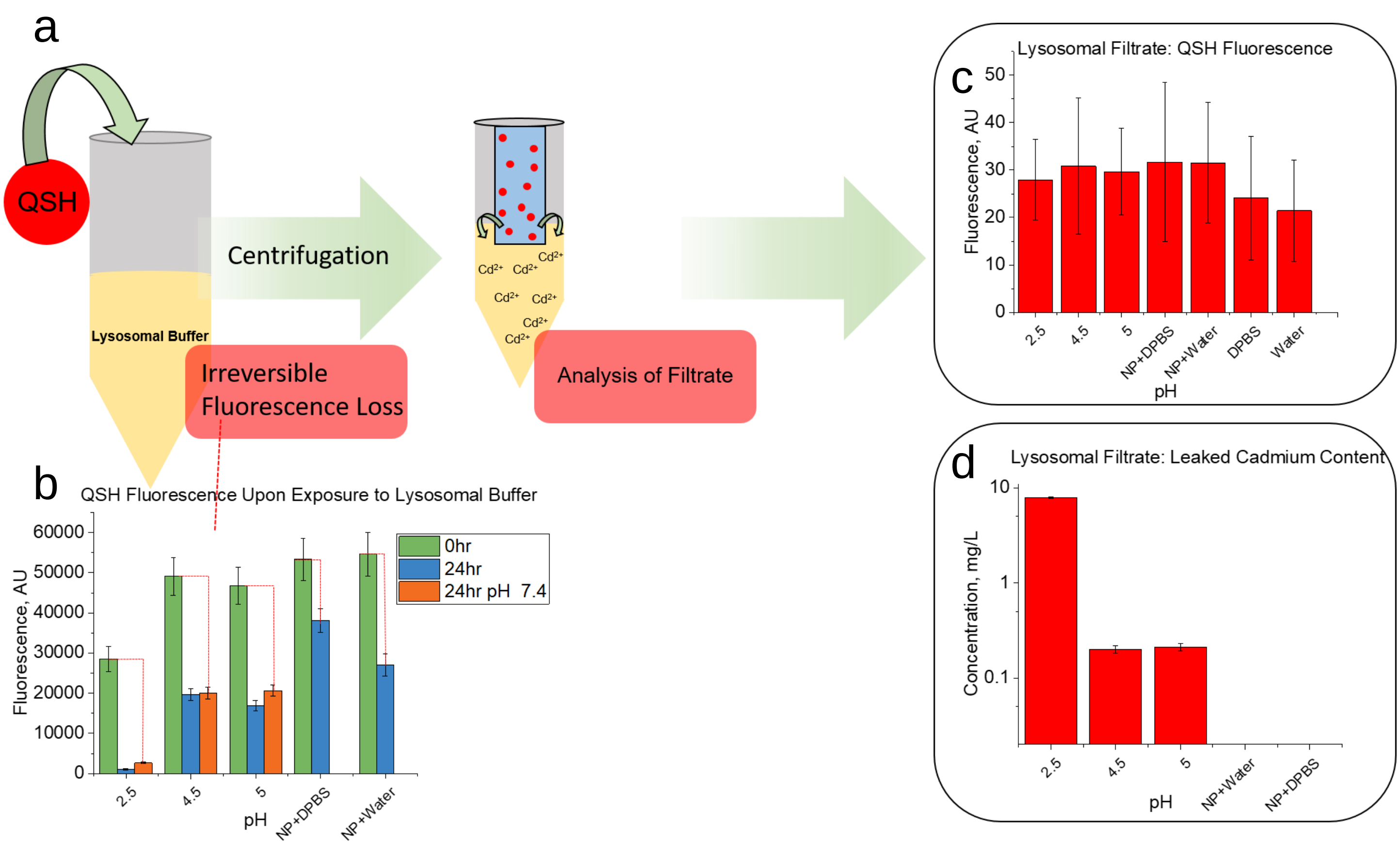

Supplementary Figure 5. Simulated lysosomal buffer QSH degradation experiment. (**a**) Schematic of process of exposure of 10nM QSH to lysosomal buffer and further purification and analysis. (**b**) Complete analysis of fluorescence signal after 0 and 24 hours of exposure. For QSH in low pH there was instant decrease in fluorescence with substantial fluorescence loss after 24 hours for all pH environments. All solutions were increased back to physiological pH of 7.4 with limited to no increase in fluorescence, which infers irreversible loss of fluorescence signal. (**c**) Filtrate analysis of all solutions show no significant QSH fluorescence in filtrate after centrifugation, indicative of limited to no intact QSH in filtrate. (**d**) However, significant cadmium content appears in lysosomal buffers, of pH 2.5, 4.5, and 5.0. No detectable free cadmium was found in filtrate with QSH exposure to DPBS and/or water.

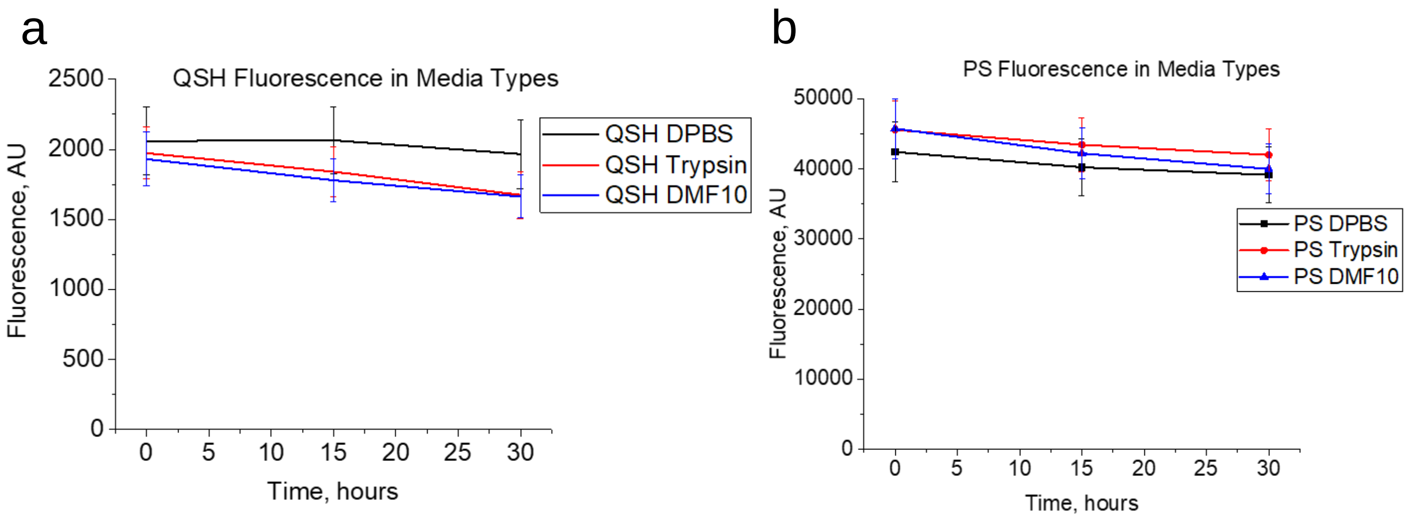

Supplementary Figure 6. Media Interference study. (**a**) Data indicate no significant difference between QSH in growth media (red) and trypsin (blue). Slight difference occurs for QSH in DPBS (black) to that in other media types. (**b**) PS showed no significant difference between all media types, and substantial stability in DPBS (black), trypsin (red), and growth media (blue).

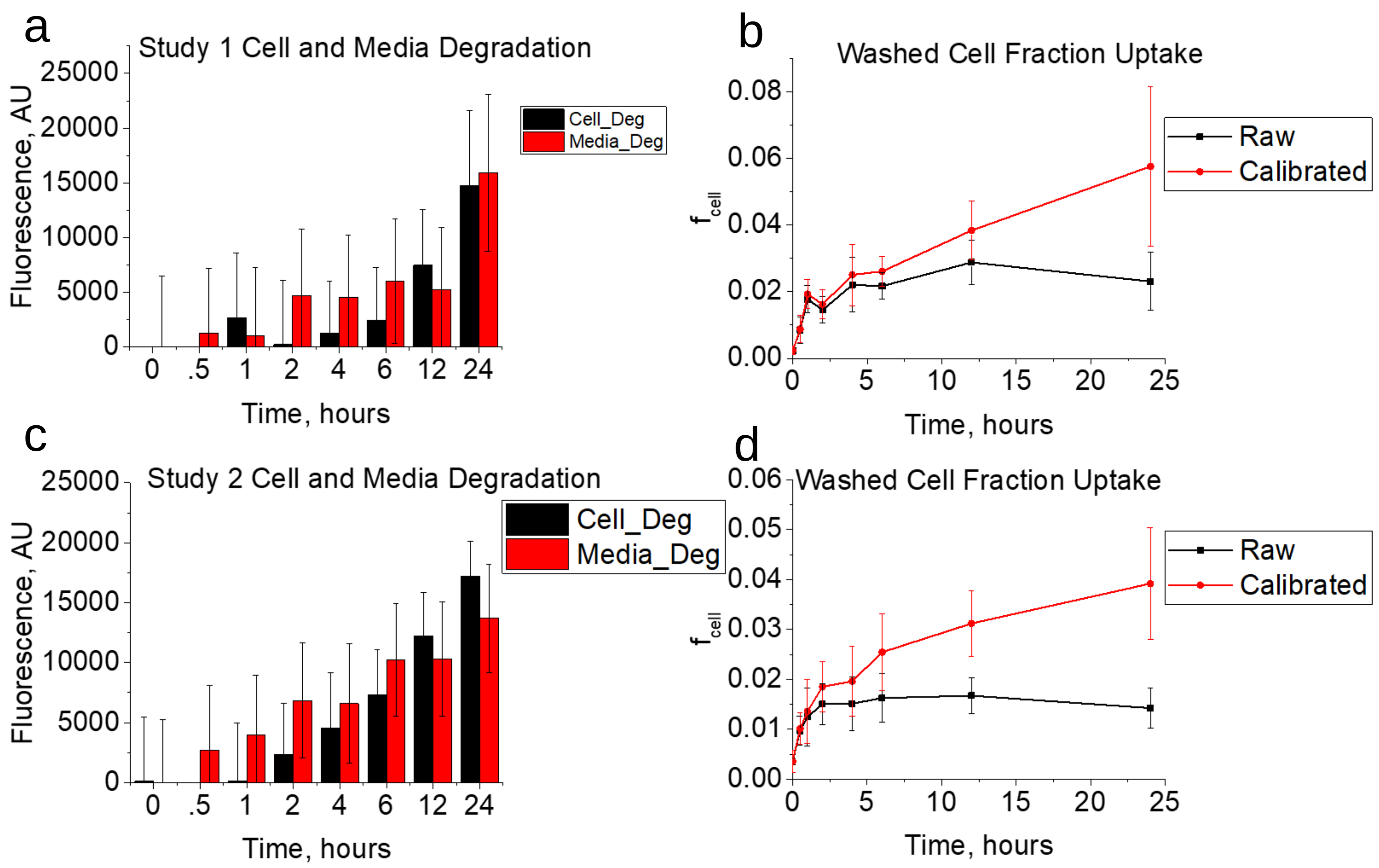

Supplementary Figure 7. Study 1 and 2 experimental data for QSH CF degradation and fraction of dose. Study 1(**a-b**) and 2 (**c-d**) datasets are the results given in Figure 2. (**a,c**) Shows raw fluorescent signatures for degradation for both studies. These values represent differences in fluorescence for CSI_t with MPE_t and MPE_0 with MPE_t for cell and media induce degradation, respectively. (**b,d**) f_{cell} values obtained from taking the intensities of CKD compartments relative to that of either CSI at time 0 or time t, depending on inclusion of degradation effect. This value was then taken to obtain [Uptake] (nM) QSH concentratios in cells according to equation 1 in main text of paper.

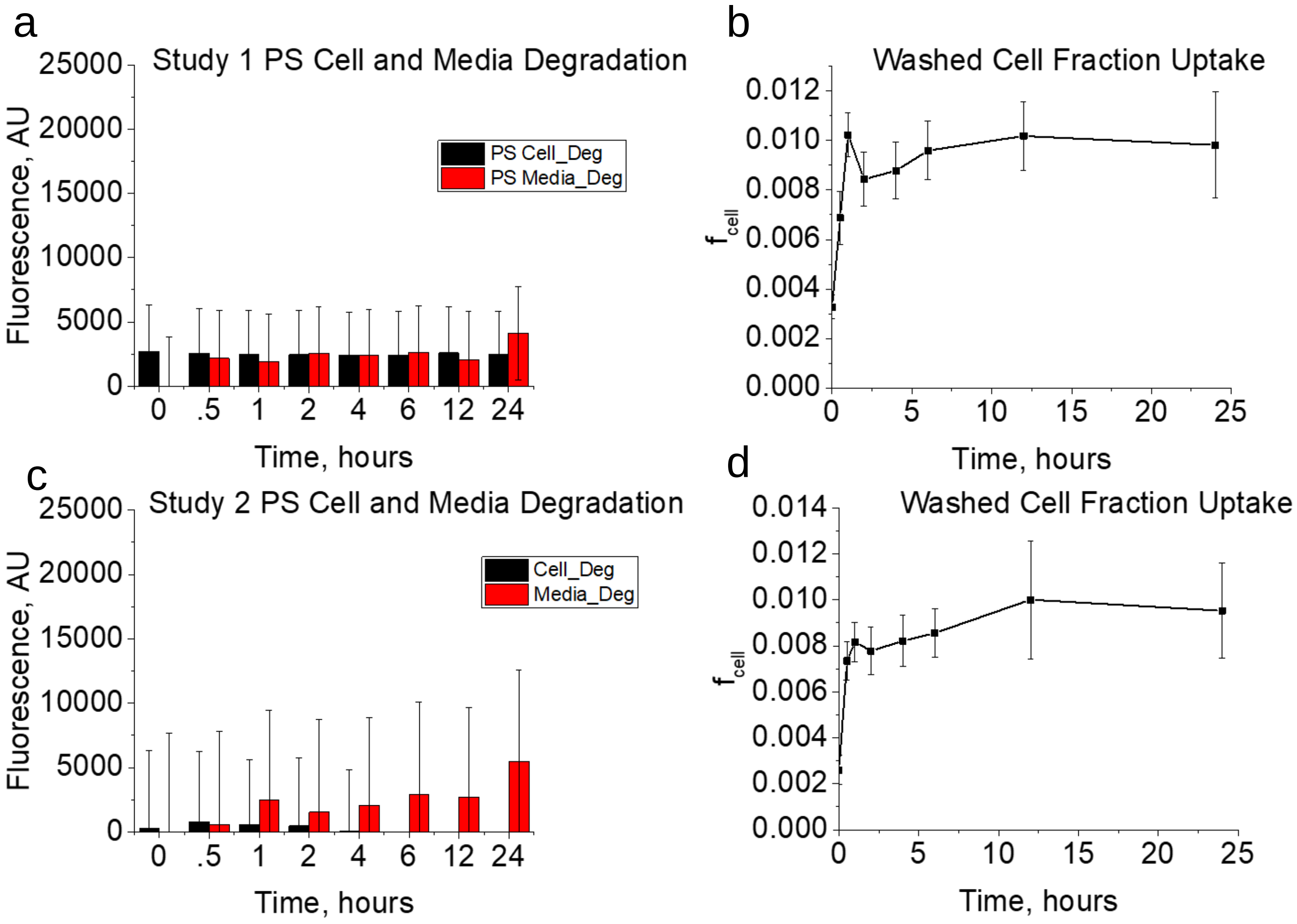

Supplementary Figure 8. Study 1 and 2 experimental data for PS CF degradation and fraction of dose**.** Study 1(**a-b**) and 2 (**c-d**) datasets are the results given in Figure 2. (**a,c**) Shows raw fluorescent signatures for degradation for both studies. These values represent differences in fluorescence for CSI_t with MPE_t and MPE_0 with MPE_t for cell and media induce degradation, respectively. (**b,d**) f_{cell} values obtained from taking the intensities of CKD compartments relative to that of either CSI at time 0 or time t, depending on inclusion of degradation effect. This value was then taken to obtain [Uptake] (nM) PS concentratios in cells according to equation 1 in main text of paper.

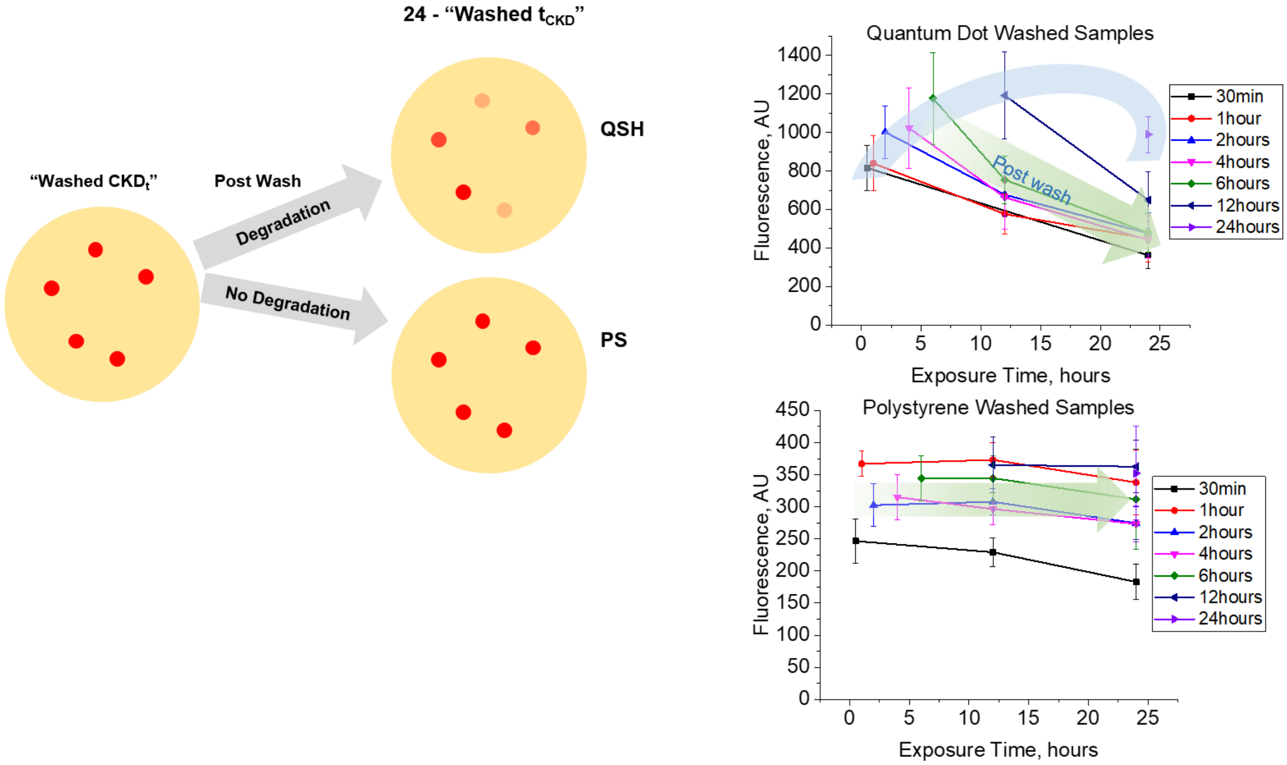

Supplementary Figure 9. Prolonged incubation of QSH and PS post-wash at time t. Here (**left**), a sample was washed at t hours and left inside incubator to determine if prolonged exposure to cellular conditions would degrade fluorescent signal over time. Quantum dot QSH, **top right**, shows significant loss in fluorescence signal after prolonged exposure to cellular environment. Polystyrene PS, **bottom right**, shows a steady maintenance of signal upon exposure to cellular environment hinting at limited to no fluorescence sensitivity to intracellular environment.

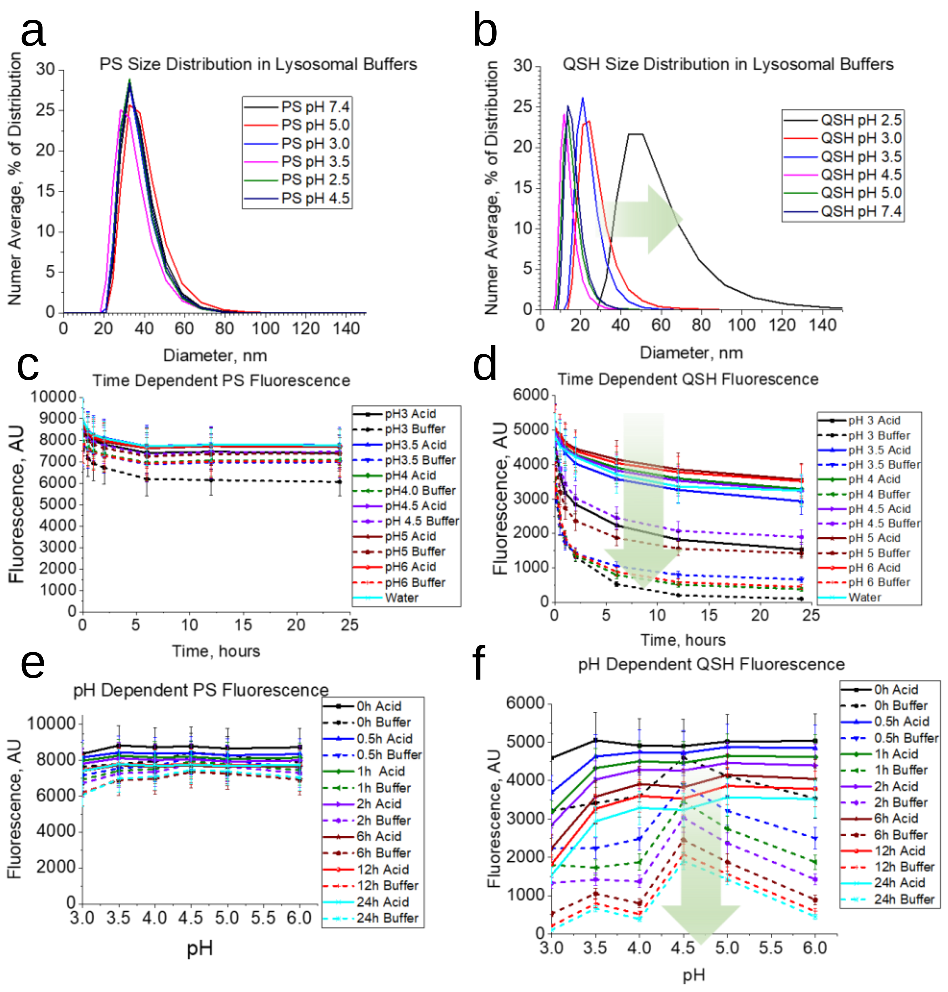

Supplementary Figure 10. Characterization after exposure to lysosomal conditions. DLS size analysis for (**a**) QSH and (**b**) PS indicate agglomeration for lower pH values for QSH and stability for PS. (**c**) Data show significant stability for PS fluorescence at 595nm emission for water and all exposure pHs under non-buffer (solid lines, Acid) and buffer (dashed lines, Buffer) conditions. However, there is a significant difference between fluorescence under buffer exposure at pH 3. (**d**) shows QSH signal at 620nm emission at all time points, with a gradual decrease over time for all pHs. (**e, f**) pH dependent studies show that (**e**) PS exhibited no significant difference between buffer and acid exposure samples under the same pH conditions and (**f**) QSH exhibited a significant difference (P<0.05) between all buffer and acid exposure samples under the same pH conditions.

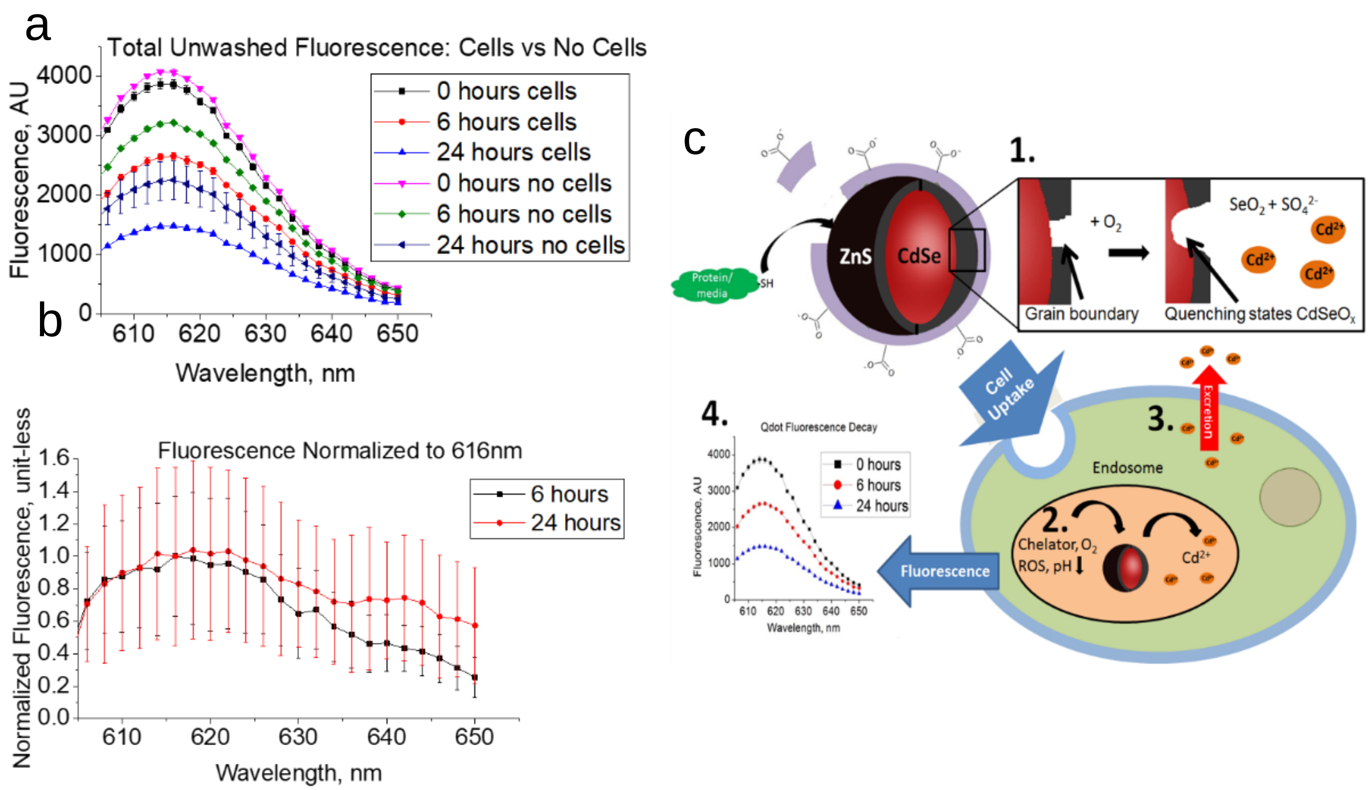

Supplementary Figure 11. Analysis of cell-induced fluorescence degradation upon exposure to cellular environment. (**a**) Full spectral analysis of unwashed wells with and without cell exposure for QSH. Washed cell fluorescence (**b**) normalized for 6 and 24 hours shows spectral broadening due to cell-induced degradation. Here, broadening shows an increase from 6 to 24 hours. (**c**) Diagram of potential mechanisms of cell-induced degradation through lysosomal sequestration with **1** surface degradation, **2** lysosomal sequestration, **3** possible cadmium leakage, and **4** fluorescence loss due to decreased pH and chelator exposure.

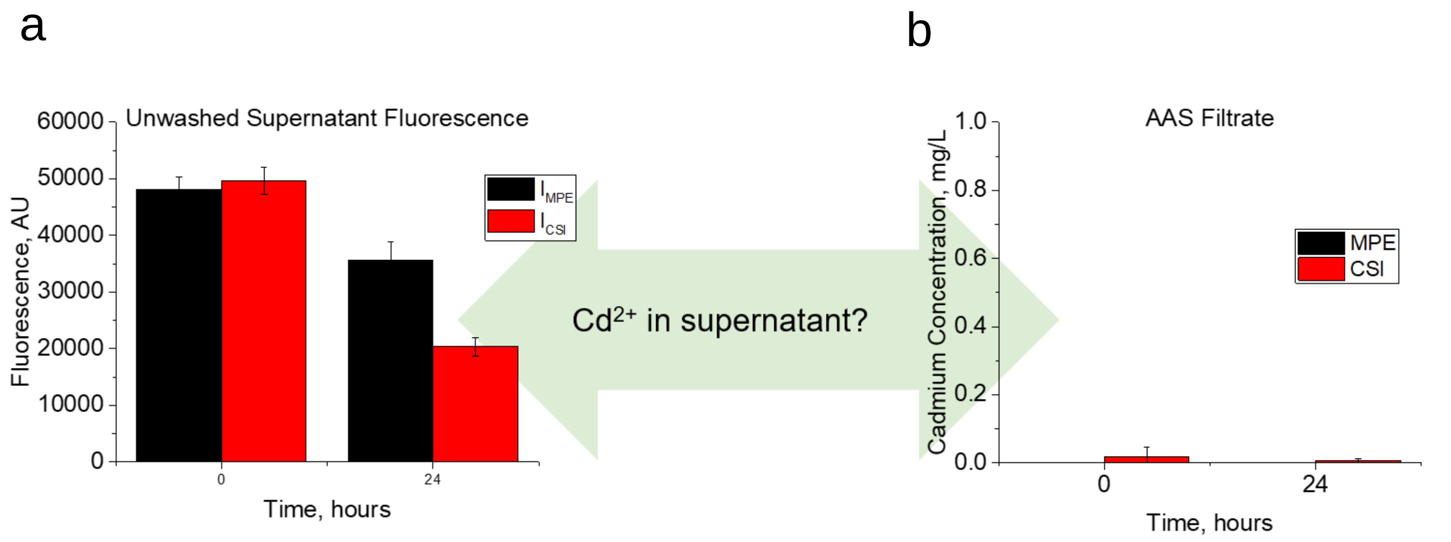

Supplementary Figure 12. Supernatant filtrate analysis for free cadmium from cell-induced degradation of QSH. (**a**) Data indicate minimal cell-induced degradation at 0 hours and significant ($P<0.05$) cell-induced degradation for 24 hours. To determine if free cadmium is present outside the cells, samples (supernatant) were centrifuged and filtrate was analyzed for cadmium content. (**b**) Here, no significant cadmium content was detected.

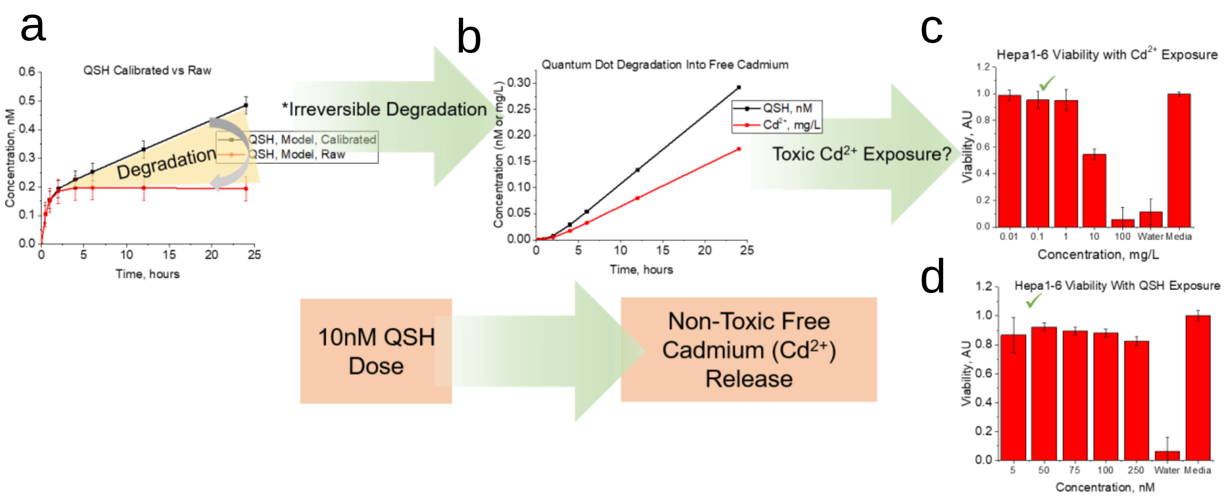

Supplementary Figure 13. Model output with QSH degraded and Cd^{2+} formation. (**a**) Model fit to calibrated datasets (black) and raw datasets (red). The difference in concentrations between datasets is described via degradation rate constant, k_{deg}, and provides additional information on concentration of degraded QSH (**b**, black) and subsequent free cadmium (Cd^{2+}) (**b**, red). The concentrations of Cd^{2+} from the degraded QSH core are not high enough to induce toxicity, as shown in (**c**) for $Cd(NO_3)_2$ exposure studies on Hepa1-6 cell line for concentrations ranging from .01-100mg/L Cd^{2+} for 24 hours. No significant toxicity is apparent for doses less than 10mg/L. (**d**) As for QSH, no toxicity is also noted for doses used in this study.

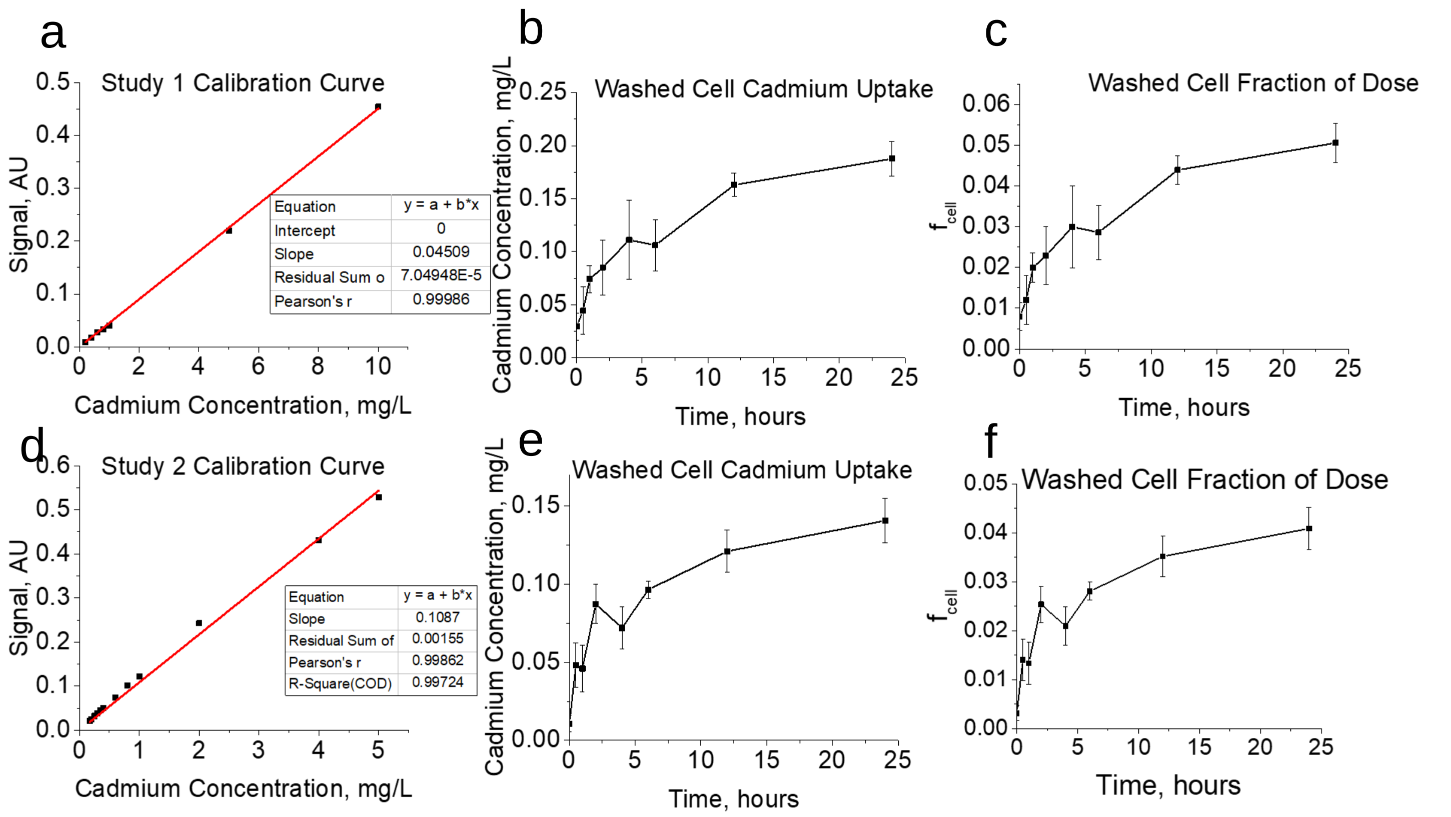

Supplementary Figure 14. Study 1 and 2 AAS Results. Study 1(**a-c**) and Study 2 (**d-f**) were run separetly approximately one (1) month apart. (**a,d**) Calibration curves used to interpolate cadmium concentration for washed cell samples taken from the CKD compartment.(**b,e**) Washed cell cadmium content obtained from CKD compartments, in mg/L over the time of their respective studies. These values were then divided by the total CSI to (**c,f**) to obtain a fractional uptake in cells.

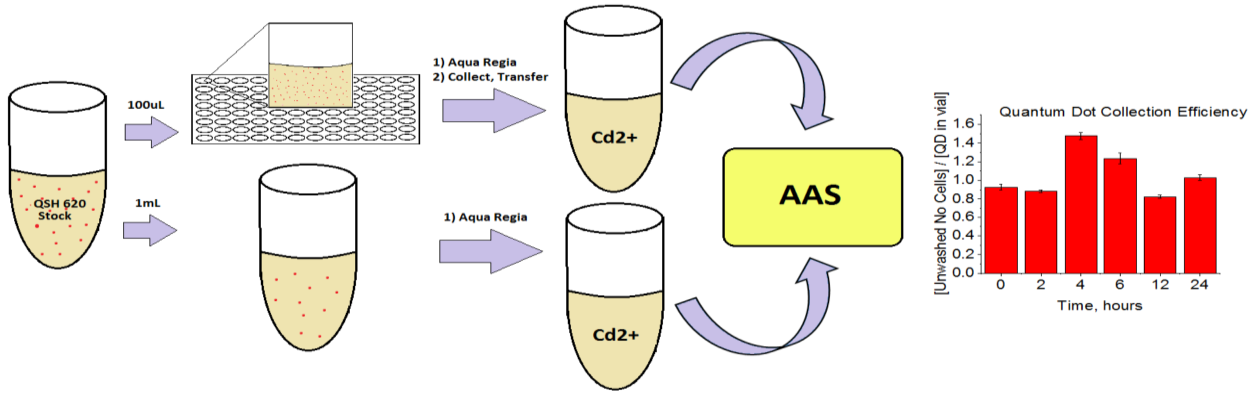

Supplementary Figure 15. Collection efficiency experiment was performed to ensure full extraction of dissolved cadmium in wells. In one case, a triplicate of wells exposed to 10nM QD from the MPE compartment was dissolved in 1/3% v/v aqua regia, while in the other, 10nM QSH was dissolved a vial with 1/3% v/v aqua regia. Both scenarios were then transferred to vials for AAS analysis.and measured for cadmium content. The ratio of MPE_t to vials were taken, as the plots indicate [Unwashed No Cells] / [QD in vial].. No significant trends in data were noted, and values ratios remained approximately equal to 1, which indicates diluted vials and collected wells were similar in cadmium content.

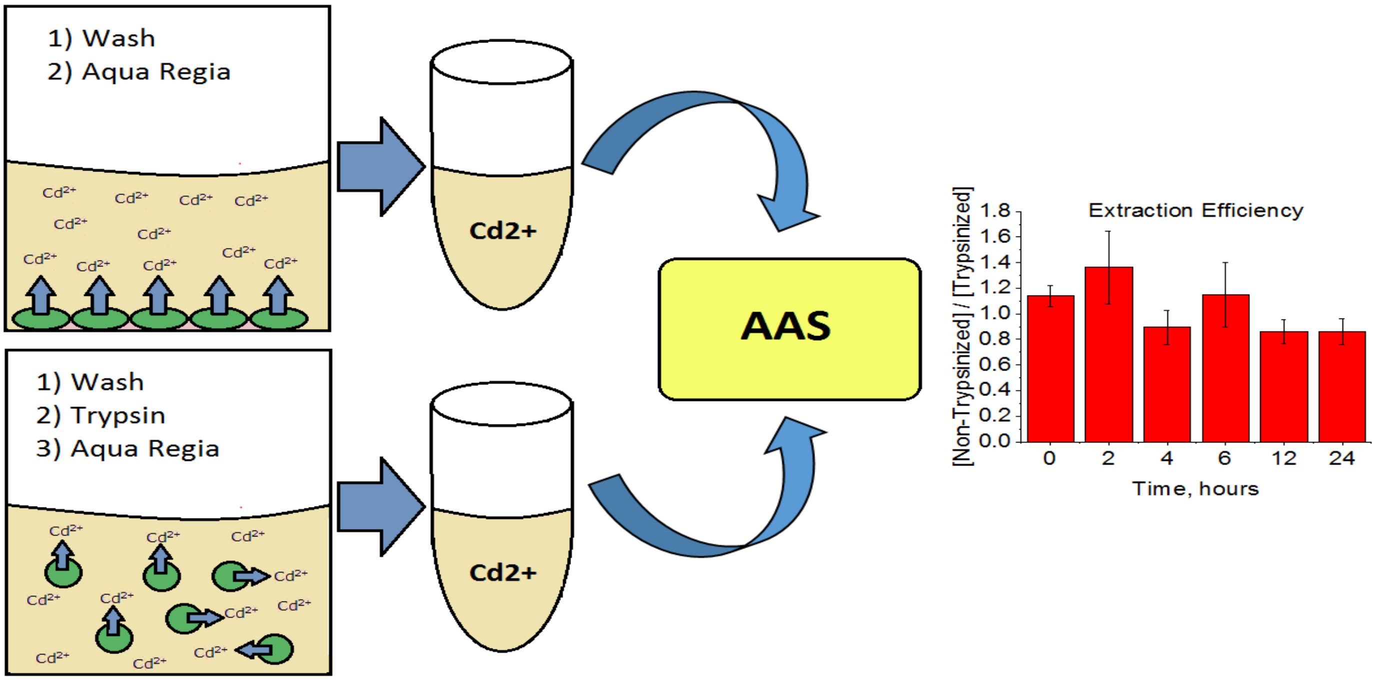

Supplementary Figure 16. Extraction efficiency for washed well cadmium content. 2X washed cells from CKD_t exposed to trypsin or no trypsin with the 10nM QSH dose were all exposed to 1/3 v/v% aqua regia for 10 minutes and transferred to vials for AAS analysis. The ratio of CKD Cd content of non-trypsinized to trypsinized was close to 1, indicative of full cadmium extraction from cell interior.

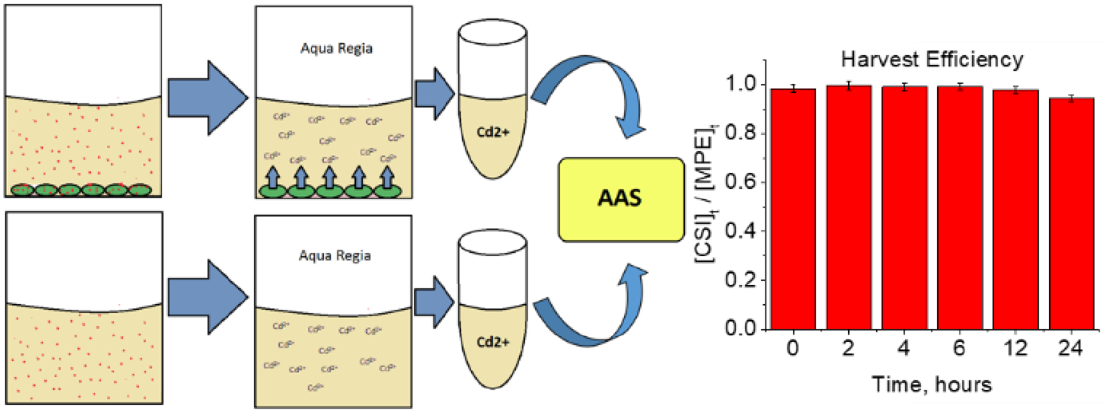

Supplementary Figure 17. Harvest efficiency above describes the potential to harvest QSH from the total well when there are cells present. Wells exposed to equal doses of QSH with and without cells (unwashed) were given equal doses of 1/3 v/v% aqua regia for 10 minutes. AAS was performed and the ratio of unwashed cells to no cells is approximately 1, indicative of full cadmium extraction, as well as minimal cellular matrix interference on the AAS instrument.

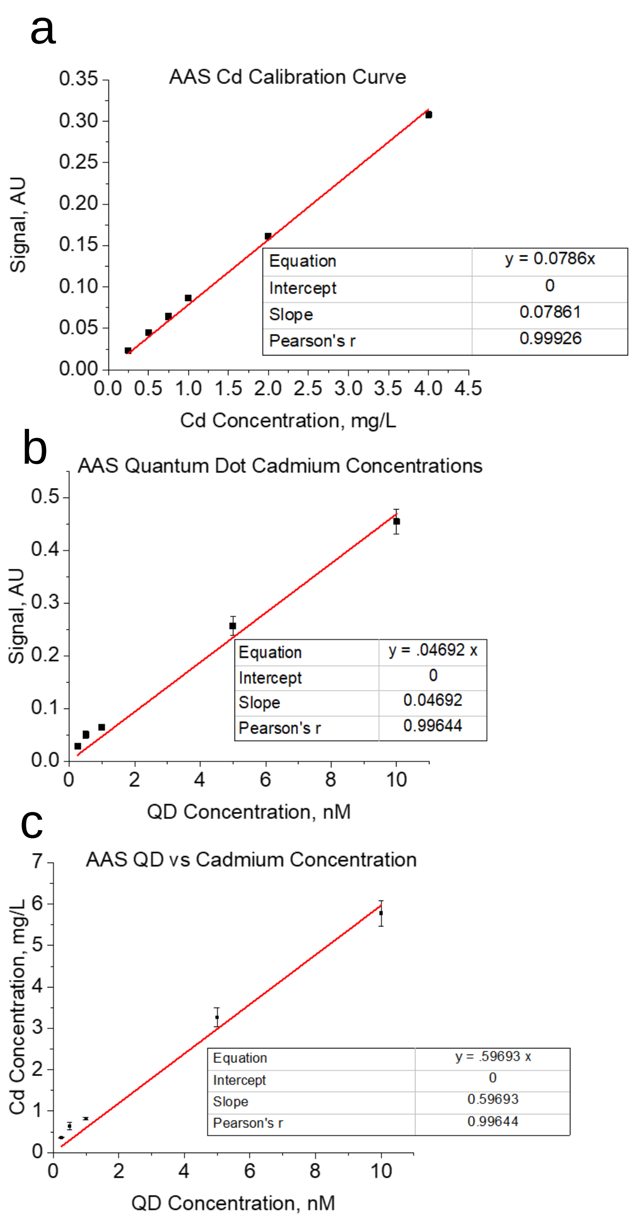

Supplementary Figure 18. AAS Calibration curve for LOD and LOQ quantitation and QD Correlation. **(a)** Cadmium concentrations showing linearity from .25 to 4.0mg/L cadmium. (**b**) Digested QSH ranging from .25 to 10nM QSH concentrations with respective signal show linearity for all concentrations shown. Slopes from (**a**) and (**b**) were used to determine correlations between QSH and cadmium concentrations (**c**) for further comparison to CF datasets.

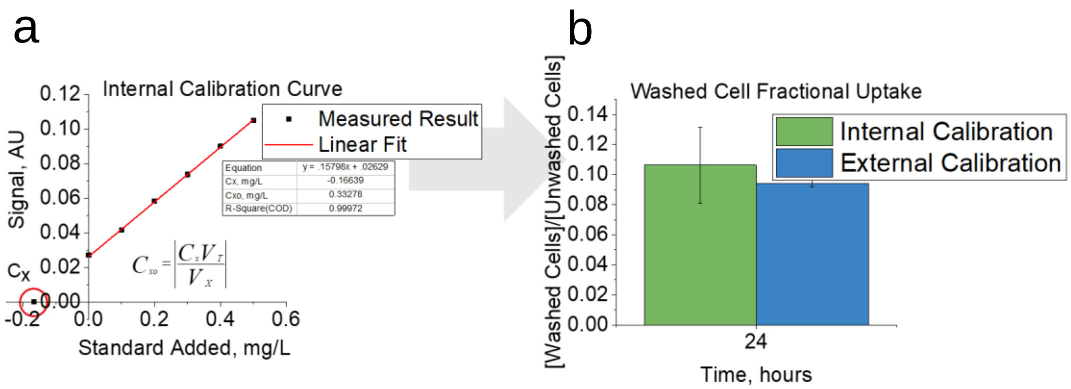

Supplementary Figure 19. Standard additions technique compared to conventional external calibration method. Comprehensive check on cell matrix interference on AAS signal was performed using an internal calibration curve (**a**) where a 24 hour sample was spiked with increasing concentrations of $Cd(NO_3)_2$ stock. The output (internal calibration) from (**a**) was compared to conventional 6-point external calibration curve data in (**b**) No significant difference exists between the two techniques, indicating limited to no cell matrix interference.

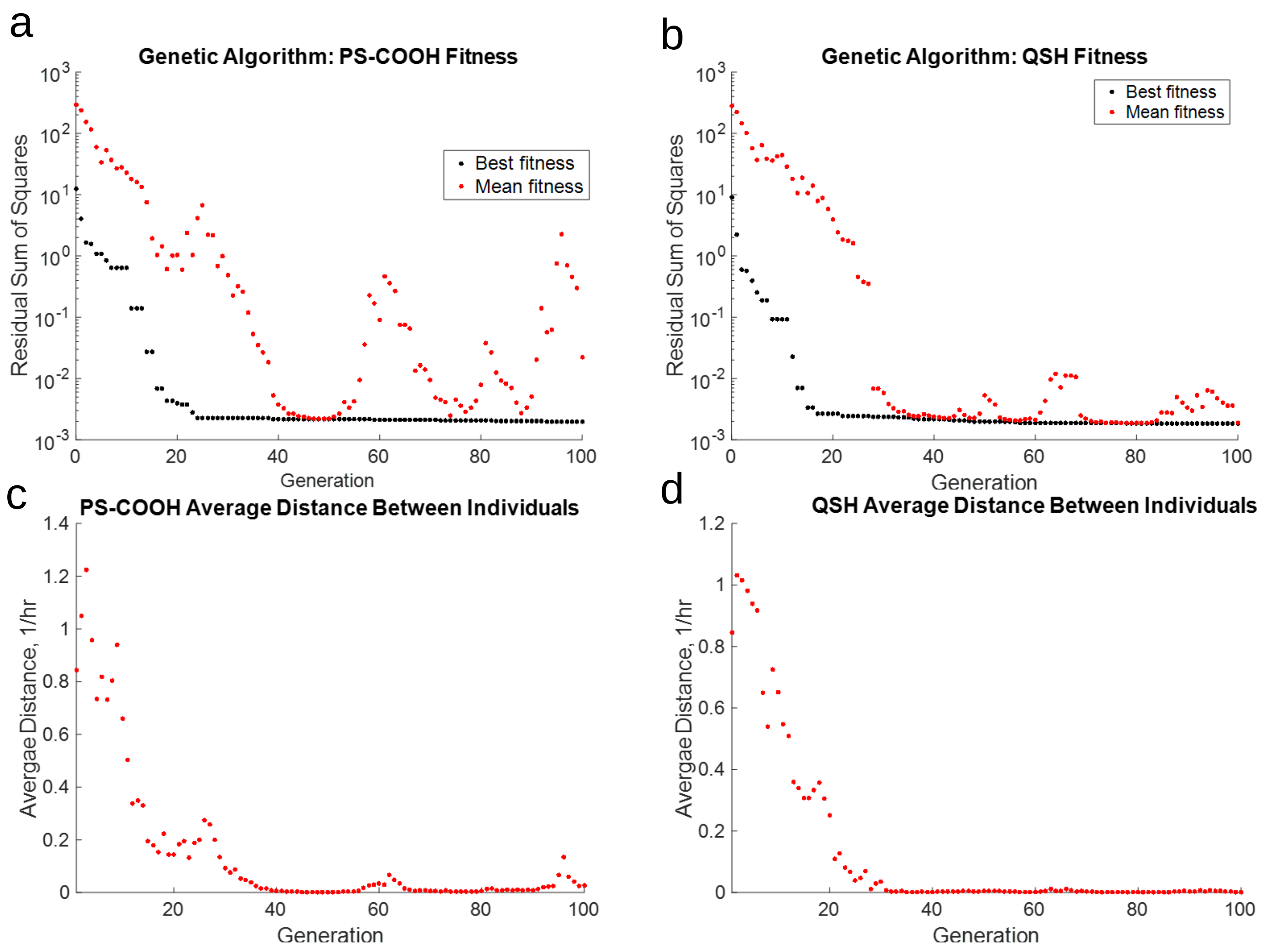

Supplementary Figure 20. Genetic algorithm output for each generation of optimization process. (**a, b**) show the fitness function (residual sum of squares) at each generation for (**a**) PS and (**b**) QSH. (**a**) shows model convergence to optimal solution of k values at approximately 25 generations (best fitness). (**b**) shows model convergence to optimal solution of k values at approximately 20 generations for QSH. Model search for additional best fit is represented by fluctuations in mean fitness (**a,b**) of population for a given generation (red, peaks). (**c, d**) show average distance between vectors of a population of 50 individuals at a given generation. As the simulation converges, the distance between vectors decreases.

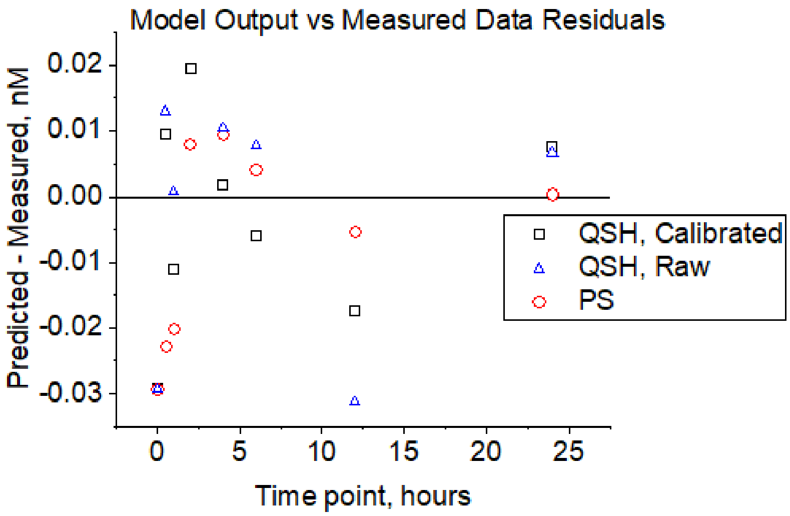

Supplementary Figure 21. Model output residuals for QSH raw and calibrated as well as PS data. Detailed residual analysis is shown for each model output difference to a particular measured concentration at time t. The apparent randomly distributed residuals around 0 indicate decent model fit to measured data.

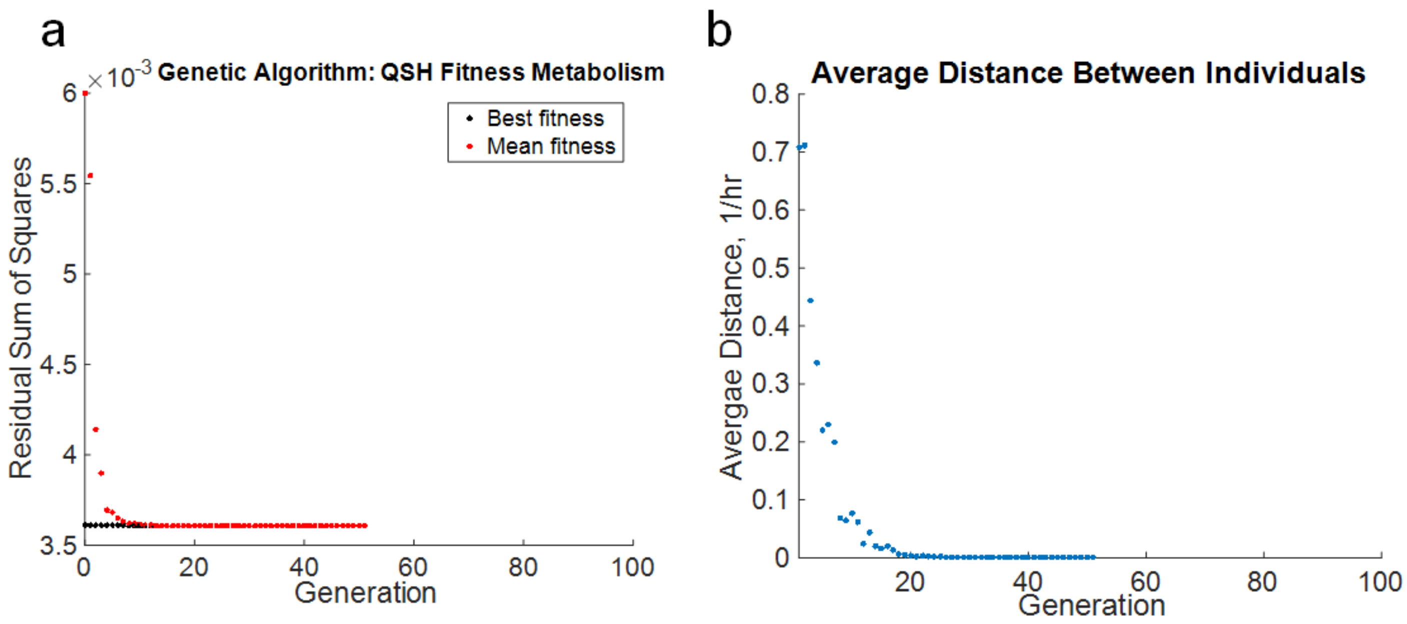

Supplementary Figure 22. Genetic algorithm output for each generation. (**a**) Fitness function (residual sum of squares) at each generation for model fit to raw QSH data. (**a**) Model convergence to optimal solution of k values at approximately 10 generations (best fitness). (**b**) Average distance between vectors of a population of 50 individuals at a given generation. As the simulation converges, the distance between vectors decreases.

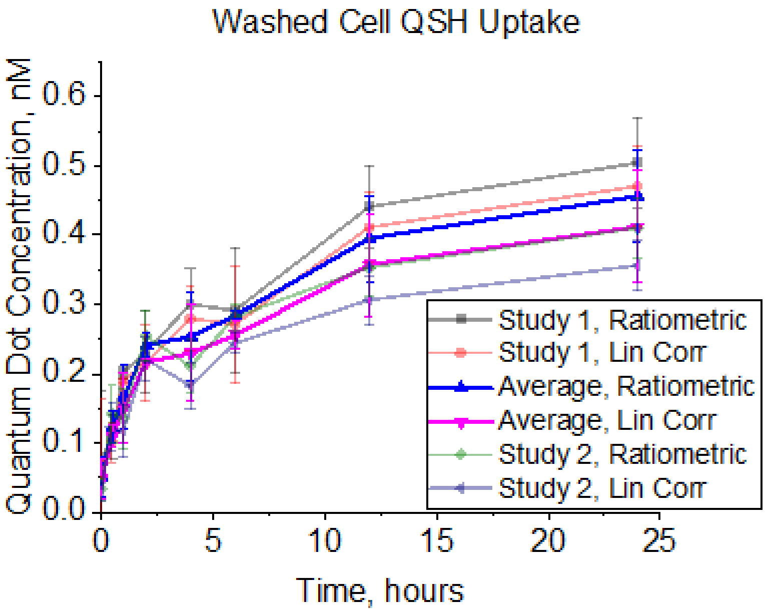

Supplementary Figure 23. Ratiometric vs Linear Correlation for AAS Raw Cadmium Concentrations in Washed CKD Samples. Here, as described in online methods, ratiometric and linear correlation techniques to translate cadmium concentrations obtained in AAS to QSH concentrations (nM) do not differ significantly (P>0.05). Thus, both techniques are considered equivalent.

Supplementary Text

Mathematical Degradation and Uptake Proof

All parameter labels (MPE, CSI, and CKD) are in reference to the diagram in **Figure 1b**. Any NM located in the CSI compartment at time t is *exposed to both media and cells*, which by default, cause the NM located in the CSI compartment to undergo total degradation (media and cell), decreasing its total intensity from its starting intensity at time 0, shown in the equation (4) below.

$$I_{CSI_t} = I_{CSI_0} - I_{deg_t} \tag{1}$$

Equation (4) can be rearranged to solve for I_{deg_t}.

$$I_{deg_t} = I_{CSI_0} - I_{CSI_t} \tag{2}$$

NM located within cells in the CKD compartment have undergone both media and cell induced degradation. Here, the raw NM intensity obtained from washed cells at time *t* for the CKD compartment (I_{CKD_t}) should be equal to the theoretical NM intensity of the washed cells *under non-degradative conditions* at time *t* for CKD ($I_{CKD}{}'_t$) minus the fraction of degradation, which is the intensity of degradation at time *t* with respect to total intensity at time 0, specifically

$$I_{CKD_t} = I_{CKD'_t} - \frac{I_{deg_t}}{I_{CSI_0}} * I_{CKD}{}'_t \tag{3}$$

Equation (6) can be rearranged to obtain the theoretical NM intensity of the washed cells under non-degradative conditions at time *t*,

$$I_{CKD}{}'_t = \frac{I_{CKD_t}}{\left(1 - \frac{I_{deg_t}}{I_{CSI_0}}\right)} \tag{4}$$

where the theoretical NM intensity of the washed cells under non-degradative conditions at time *t* , ($I_{CKD}{}'_t$) can be used to obtain the *calibrated fraction of NM uptake* by cells under non-degradative conditions by dividing by the initial total unwashed fluorescence at time 0.

$$f_{cell}{}' = \frac{I_{CKD'_t}}{I_{CSI_0}} \tag{5}$$

The calibrated fraction of NM uptake by cells under degradative (assuming time in intracellular environment induces degradation) conditions is equal to the raw intensity of washed cells in CKD compartment taken relative to raw intensity of unwashed cells in CSI compartment at time *t*. *Here, we assume NM located within CKD and CSI compartments have undergone media and cell-induced degradation, giving it a calibration for this effect*.

$$f_{cell_c} = \frac{I_{CKD_t}}{I_{CSI_t}} \tag{6}$$

Assuming that the internal standard (I_{CSI_t}) and washed cells in CKD (I_{CKD_t}) undergo degradation under the same conditions, the fraction here,f_{cell_c}, should be equal to the fraction of uptake under non-degradative conditions ($f_{cell}{}'$),

$$f_{cell}{}' = f_{cell_c} \tag{7}$$

Equation (7) can be rearranged by substituting their respective intensities according to equations (5) and (6),

$$\frac{I_{CKD'_t}}{I_{CSI_0}} = \frac{I_{CKD_t}}{I_{CSI_t}} \tag{8}$$

where I_{CKD_t} can be substituted by equation (3) above. This is shown by equation (9) below:

$$\frac{I_{CKD'_t}}{I_{CSI_0}} = \frac{I_{CKD'_t} - \frac{I_{deg_t}}{I_{CSI_0}} * I_{CKD'_t}}{I_{CSI_t}} \tag{9}$$

Equation (9) can be rearranged to yield

$$I_{CKD'_t} = \frac{I_{CSI_0} I_{CKD'_t} - I_{deg_t} * I_{CKD'_t}}{I_{CSI_t}} \tag{10}$$

where I_{CSI_t} can be solved for,

$$I_{CSI_t} = \frac{I_{CSI_0} I_{CKD'_t} - I_{deg_t} * I_{CKD'_t}}{I_{CKD'_t}} \tag{11}$$

cancelling $I_{CKD'_t}$ to yield

$$I_{CSI_t} = I_{CSI_0} - I_{deg_t} \tag{12}$$

Equation (12) states that the total unwashed NM intensity after a period of time t is equal to the unwashed initial NM intensity (where we assume 0 hours of cell exposure to have no degradation) minus the intensity value of degradation for that period of time t. Substituting $(I_{CSI_0} - I_{deg})$ by the definition of equation (2), yields

$$I_{CSI_t} = I_{CSI_t} \tag{13}$$

Calculation for raw fraction of uptake of for cells is similar to the calibrated, but instead, we take it relative to $I_{CSI_0}$for comparison,

$$f_{cell_r} = \frac{I_{CKD_t}}{I_{CSI_0}} \tag{14}$$

Here, we are taking the raw CKD washed cell NM intensity (which undergoes cell and media degradation) relative to unwashed CSI compartment (which *does not* undergo cell and media degradation) to understand the degradative effect cells and media can have on a NM.

Calibrated (f_{cell_c}) and raw (f_{cell_r}) fraction of uptake for cells were then used to obtain [Uptake]$_t$ concentrations in nM at time t, according to

$$[Uptake]_t = f_{cell_x} * [Dose] \tag{15}$$

Where f_{cell_x} is f_{cell_c} or f_{cell_r} depending on if cell-induced degradation is present or not, and [Dose] is the exposure dose to cells in nM.
